# Synergies and prospects for early resolution of the neutrino mass ordering

**DOI:** 10.1038/s41598-022-09111-1

**Published:** 2022-03-30

**Authors:** Anatael Cabrera, Yang Han, Michel Obolensky, Fabien Cavalier, João Coelho, Diana Navas-Nicolás, Hiroshi Nunokawa, Laurent Simard, Jianming Bian, Nitish Nayak, Juan Pedro Ochoa-Ricoux, Bedřich Roskovec, Pietro Chimenti, Stefano Dusini, Mathieu Bongrand, Rebin Karaparambil, Victor Lebrin, Benoit Viaud, Frederic Yermia, Lily Asquith, Thiago J. C. Bezerra, Jeff Hartnell, Pierre Lasorak, Jiajie Ling, Jiajun Liao, Hongzhao Yu

**Affiliations:** 1 APC, CNRS/IN2P3, CEA/IRFU, Observatoire de Paris, Sorbonne Paris Cité University, 75205 Paris Cedex 13, France; 2 IJCLab, Université Paris-Saclay, CNRS/IN2P3, 91405 Orsay, France; 3 Department of Physics and Astronomy, University of California at Irvine, Irvine, CA 92697 USA; 4 LNCA Underground Laboratory, CNRS/IN2P3-CEA, Chooz, France; 5 Departamento de Física, Universidade Estadual de Londrina, Londrina, PR 86051-990 Brazil; 6 INFN, Sezione di Padova, via Marzolo 8, 35131 Padua, Italy; 7 Institute of Particle and Nuclear Physics, Faculty of Mathematics and Physics, Charles University, V Holešovičkách 2, 180 00 Prague 8, Czech Republic; 8 Department of Physics, Pontifícia Universidade Católica do Rio de Janeiro, Rio de Janeiro, RJ 22451-900 Brazil; 9 SUBATECH, CNRS/IN2P3, Université de Nantes, IMT-Atlantique, 44307 Nantes, France; 10 Department of Physics and Astronomy, University of Sussex, Falmer, Brighton, BN1 9QH UK; 11 Sun Yat-sen University, NO. 135 Xingang Xi Road, Guangzhou, 510275 China

**Keywords:** Experimental particle physics, Phenomenology

## Abstract

The measurement of neutrino mass ordering (MO) is a fundamental element for the understanding of leptonic flavour sector of the *Standard Model of Particle Physics.* Its determination relies on the precise measurement of $\Delta m^2_{31}$ and $\Delta m^2_{32}$ using either neutrino *vacuum oscillations*, such as the ones studied by medium baseline reactor experiments, or *matter effect modified oscillations* such as those manifesting in long-baseline neutrino beams (LB$\nu$B) or atmospheric neutrino experiments. Despite existing MO indication today, a fully resolved MO measurement ($\ge 5\sigma$) is most likely to await for the next generation of neutrino experiments: JUNO, whose stand-alone sensitivity is $\sim 3\sigma$, or LB$\nu$B experiments (DUNE and Hyper-Kamiokande). Upcoming atmospheric neutrino experiments are also expected to provide precious information. In this work, we study the possible context for the earliest full MO resolution. A firm resolution is possible even before 2028, exploiting mainly vacuum oscillation, upon the combination of JUNO and the current generation of LB$\nu$B experiments (NOvA and T2K). This opportunity is possible thanks to a powerful synergy boosting the overall sensitivity where the sub-percent precision of $\Delta m^2_{32}$ by LB$\nu$B experiments is found to be the leading order term for the MO earliest discovery. We also found that the comparison between *matter* and *vacuum* driven oscillation results enables unique discovery potential for physics beyond the Standard Model.

## Introduction

The discovery of the *neutrino* ($\nu$) *oscillations* phenomenon has completed a remarkable scientific endeavor lasting several decades changing forever our understanding of the leptonic sector’s phenomenology of the *standard model of elementary particles* (SM). The new phenomenon was taken into account by introducing massive neutrinos and consequently neutrino flavour mixing and the possibility of violation of charge conjugation parity symmetry or CP-violation (CPV); e.g., review^1^.

Neutrino oscillations imply that the neutrino mass eigenstates (${\nu _1}$, ${\nu _2}$, ${\nu _3}$) spectrum is non-degenerate, so at least two neutrinos are massive. Each mass eigenstate (${\nu _i}$; with *i* = 1, 2, 3) can be regarded as a non-trivial mixture of the known neutrino flavour eigenstates (${\nu _e}$, ${\nu _\mu }$, ${\nu _\tau }$), linked to the three (*e*, $\mu$, $\tau$) respective charged leptons. Since no significant experimental evidence beyond three families exists so far, the mixing is characterised by the $3 \times 3$ so called *Pontecorvo-Maki-Nakagawa-Sakata* (PMNS)^2,3^ matrix, assumed to be unitary, thus parameterised by three independent mixing angles (${\theta _{12}}$, ${\theta _{23}}$, ${\theta _{13}}$) and one CP phase ($\delta _{\tiny \text {CP}}$). The neutrino mass spectra are indirectly known via the two measured *mass squared differences*, indicated as $\delta m^2_{21}$($\equiv m_2^2-m_1^2$) and $\Delta m^2_{32}$ ($\equiv m_3^2-m_2^2$), respectively, related to the ${\nu _2}$/${\nu _1}$ and ${\nu _3}$/${\nu _2}$ pairs. The neutrino absolute mass is not directly accessible via neutrino oscillations and remains unknown, despite considerable active research^4^.

As of today, the field is well established both experimentally and phenomenologically. All relevant parameters (${\theta _{12}}$, ${\theta _{23}}$, ${\theta _{13}}$ and $\delta m^2_{21}$, $|\Delta m^2_{32}|$) are known to the few percent precision. The $\delta _{\tiny \text {CP}}$ phase and the sign of $\Delta m^2_{32}$, the so-called Mass Ordering (MO), remain unknown despite existing hints (i.e., $<3\sigma$ effects). CPV processes arise if $\delta _{\tiny \text {CP}}$ is different from 0 or $\pm \pi$, i.e., CP-conserving solutions. The measurement of the MO has the peculiarity of having only a binary solution, either normal mass ordering (NMO), in case $\Delta m^2_{31} >0$, or inverted mass ordering (IMO) if $\Delta m^2_{31} <0$. In order words, determining MO implies to know which is the lightest neutrino ${\nu _1}$ (or ${\nu _3}$), respective the case of NMO (IMO). The positive sign of $\delta m^2_{21}$ is known from solar neutrino data^5–9^ combined with KamLAND^10^, establishing the solar large mixing angle MSW^11,12^ solution.

## Mass ordering knowledge

This publication focuses on the global strategy to achieve the earliest and most robust MO determination scenario. MO has rich implications not only for the terrestrial oscillation experiments, to be discussed in this paper, but also for non-oscillation experiments like search for neutrinoless double beta decay (e.g., review^13^) or from more broad aspects, from a fundamental theoretical (e.g., review^14^), an astrophysical (e.g., review^15^), and cosmological (e.g., review^16^) points of view. Present knowledge from global data^4,17–19^ implies a few $\sigma$ hints on both MO and $\delta _{\tiny \text {CP}}$, where the latest results were reported at *Neutrino 2020 Conference*^20^. According to the latest NuFit5.0^21^ global data analysis, NMO is favoured up to $2.7\sigma$. However, this preference remains fragile, as it will be explained later on.

Experimentally, MO can be addressed via three very different techniques (e.g.,^22^ for earlier work): (a) medium baseline reactor experiment^23^ (i.e., JUNO) (b) long-baseline neutrino beams (labeled here LB$\nu$B) and (c) atmospheric neutrino based experiments. MO determination by LB$\nu$B and atmospheric neutrinos relies on *matter effects*^11,12^ as neutrinos traverse the Earth over long enough baselines. Since Earth is made of matter, and not of anti-matter, the effect of elastic forward scattering for electron anti-neutrinos and neutrinos depends on the sign of $\Delta m^2_{32}$. Instead, JUNO^24^ is currently the only experiment able to resolve MO via dominant *vacuum* oscillations [JUNO has a minor matter effect impact, mainly on the $\delta m^2_{21}$ oscillation while tiny on MO sensitive $\Delta m^2_{32}$ oscillation^25^], thus holding a unique insight and capability in the MO world strategy.

The current generation of LB$\nu$B experiments, here called LB$\nu$B-II [The first generation LB$\nu$B-I are here considered to be K2K^26^, MINOS^27^ and OPERA^28^ experiments], are NOvA^29^ and T2K^30^. These are to be followed up by the next generation LB$\nu$B-III with the DUNE^31^ and the Hyper-Kamiokande (HK)^32^ experiments, which are expected to start taking data around 2027. In Korea, a possible second HK detector would enhance its MO determination sensitivity^33^. In this paper we focus mainly on the immediate impact of the LB$\nu$B-II. Nonetheless, we shall highlight the prospect contributions by LB$\nu$B-III, due to their leading order implications to the MO resolution. Contrary to those experiments, JUNO relies on high precision reactor neutrino spectral analysis for the extraction of MO sensitivity.

The relevant atmospheric neutrino experiments are Super-Kamiokande^34^ (SK) and IceCube^35^ (both running) as well as future specialised facilities such as INO^36^, ORCA^37^ and PINGU^38^. The advantage of atmospheric neutrinos experiments to probe many baselines simultaneously, is partially compensated by the more considerable uncertainties in baseline and energy reconstruction and limited $\nu /{\bar{\nu }}$ separation. The HK experiment may also offer critical MO insight via atmospheric neutrinos.

Despite their different MO sensitivity potential and time schedules (discussed in the end), it is worth highlighting each technique’s complementarity as a function of the relevant neutrino oscillation unknowns. The MO sensitivity of atmospheric experiments depends heavily on the so called $\theta _{23}$ *octant ambiguity* [This implies the approximate degeneracy of oscillation probabilities for the cases between $\theta _{23}$ and $(\pi /4-\theta _{23})$]^39^, while LB$\nu$B experiments exhibit a smaller dependence. JUNO is, however, independent, a unique asset. Regarding the unknown $\delta _{\tiny \text {CP}}$, its role in atmospheric and LB$\nu$B’s inverts, while JUNO remains uniquely independent. This way, the MO sensitivity dependence on $\delta _{\tiny \text {CP}}$ is less important for atmospheric neutrinos (i.e. washed out), but LB$\nu$B-II are to a great extent handicapped by the degenerate phase-space competition to resolve both $\delta _{\tiny \text {CP}}$ and MO simultaneously. In brief, the MO sensitivity interval of ORCA/PINGU swings about the 3$\sigma$ to 5$\sigma$, depending on the value of $\theta _{23}$  and LB$\nu$B-II sensitivities are effectively blinded to MO for more than half of the $\delta _{\tiny \text {CP}}$ phase-space. However, DUNE has the unique ability to resolve MO, also via matter effects, regardless of $\delta _{\tiny \text {CP}}$. Although not playing an explicit role, the constraint on $\theta _{13}$, from reactor experiments (i.e. Daya Bay^40^, Double Chooz^41^ and RENO^42^), is critical for the MO (and $\delta _{\tiny \text {CP}}$) quest for JUNO and LB$\nu$B experiments.

This publication aims to illustrate, and numerically demonstrate, via a simplified estimation, the relevant ingredients to reach a fully resolved (i.e., $\ge 5\sigma$) MO measurement strategy relying, whenever possible, only on existing (or imminently so) experiments to yield the fastest timeline [the timelines of experiments are involved, as the construction schedules may delay beyond the scientific teams’ control. Our approach aims to provide minimal timing information to contextualise the experiments, but variations may be expected]. Our approach relies on the latest 3$\nu$ global data information^21^, summarised in Table 1, to tune our analysis to the most probable and up to date measurements on $\theta _{23}$, $\delta _{\tiny \text {CP}}$ and $\Delta m^2_{32}$, using only the LB$\nu$B inputs, as motivated later. This work updates and expands previous works^43–45^ basing the calculations on $\Delta m^2_{32}$, instead of $\Delta m^2_{\mu \mu }$, as well as including the effects of the uncertainties on the relevant oscillation parameters. In addition, the here presented results are contextualized in the current experimental landscape, in terms of current precision of the oscillation parameters and the present-day performances of current and near future neutrino oscillation experiments, providing an important insight into the prospects for solving the neutrino mass ordering.

**Table 1:** In this work, the neutrino oscillation parameters are reduced to the latest values obtained in the NuFit5.0^21^, where $\Delta m^2_{32}$, $\sin ^2 \theta _{23}$ and $\delta _{\tiny \text {CP}}$ (last two rows) were obtained by using only LB$\nu$B experiments by fixing $\delta m^2_{21}$, $\sin ^2 \theta _{12}$ and $\sin ^2 \theta _{13}$ to the values shown in this table (second row).

NuFit5.0	$\delta m^2_{21}$	$\sin ^2 \theta _{12}$	$\sin ^2 \theta _{13}$
Both MO	$7.42\times 10^{-5}$ $\hbox {eV}^2$	0.304	0.0224
LB$\nu$B	$\Delta m^2_{32}$	$\sin ^2 \theta _{23}$	$\delta _{\tiny \text {CP}}$
NMO	$2.411\times 10^{-3} \hbox {eV}^2$	0.565	$-0.91\pi$
IMO	$-2.455\times 10^{-3} \hbox {eV}^2$	0.568	$-0.46\pi$

We also aim to highlight some important redundancies across experiments that could aid the robustness of the MO resolution and exploit—likely for the first time—the MO measurements for high precision scrutiny of the standard 3$\nu$ flavour scheme. In this context, MO exploration might open the potential for manifestations of physics beyond the Standard Model (BSM), e.g., see reviews^24,46^. Our simplified approach is expected to be improvable by more complete developments (i.e. full combination of experiments’ data), once data is available. Such approach, though, is considered beyond our scope as it is unlikely to significantly change our findings and conclusions, given the data precision available today. To better accommodate our approach’s known limitations, we have intentionally performed a conservative rationale. We shall elaborate on these points further during the discussion of the final results.

## Mass ordering resolution analysis

Our analysis relies on a simplified combination of experiments able to yield MO sensitivity intrinsically (i.e. standalone) and via inter-experiment synergies, where the gain may be direct or indirect. The indirect gain implies that the sensitivity improvement occurs due to the combination itself; i.e. hence not accessible to neither experiment alone but caused by the complementary nature of the different experiments’ observables. These effects will be carefully studied, including the delicate arising dependencies to ensure accurate prediction are obtained. The existing synergies found embody a framework for powerful sensitivity boosting to yield MO resolution upon combination. To this end, we shall combine the running LB$\nu$B-II experiments with the shortly forthcoming JUNO. The valuable additional information from atmospheric experiments will be considered qualitatively, for simplicity, only at the end during the discussion of results. Unless otherwise stated explicitly, throughout this work, we shall use only the NuFit5.0^21^ best-fit values summarised in Table 1, to guide our estimations and predictions by today’s data.

### Mass ordering resolution power in JUNO

**Figure 1:**
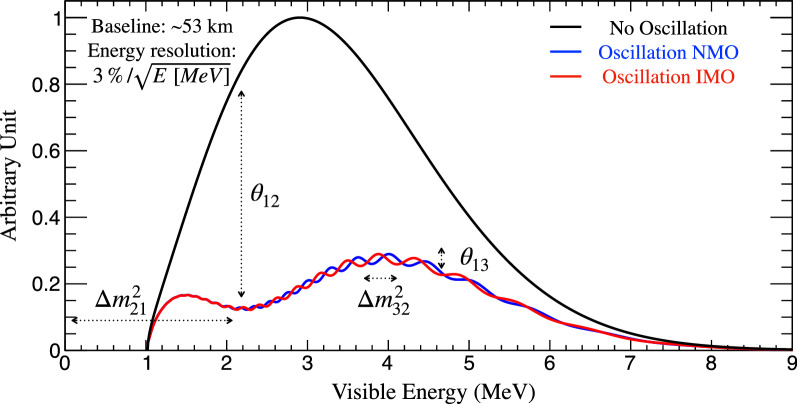
JUNO neutrino bi-oscillation spectral distorsion. JUNO was designed to exploit the spectral distortions from two oscillations simultaneously manifesting via reactor neutrinos in a baseline of $\sim$53 km. $\theta _{12}$ and $\delta m^2_{21}$ drive the slow and large amplitude ($\sin ^2 ( 2\theta _{12})/2 \approx$ 42%) disappearance oscillation with a minimum at $\sim$2 MeV visible energy. The fast and smaller amplitude ($\sin ^2 (2\theta _{13})/2 \approx$ 5%) disappearance oscillation is driven by $\theta _{13}$ and $\Delta m^2_{32}$ instead. The $\theta _{13}$ oscillation frequency pattern depends on $\Delta m^2_{32}$’s sign, thus directly sensitive to mass ordering (MO) via only *vacuum* oscillations. JUNO’s high statistics allow shape-driven neutrino oscillation parameter extraction, with minimal impact from rate-only systematics. Hence, high precision is possible without permanent reactor flux monitoring, often referred to as *near detector*(s). JUNO’s shape analysis relies on the reactor reference spectrum’s excellent control, implying high resolution, energy scale control, and a robust data-driven reference spectrum obtained with TAO^47^, a satellite experiment of JUNO. The here presented plot is for illustration purposes and the neutrino oscillation parameters are taken from NuFit5.0 (Table 1).

The JUNO experiment^24^ is one of the most powerful neutrino oscillation high precision machines. The JUNO spectral distortion effects are described in Fig. 1, and its data-taking is expected to start in 2023^48^. The possibility to explore precision neutrino oscillation physics with an intermediate baseline reactor neutrino experiment was first pointed out in^49^. Indeed JUNO alone can yield the most precise measurements of $\theta _{12}$, $\delta m^2_{21}$ , and $|\Delta m^2_{32}|$, at the sub-percent precision^48^ for the first time. Therefore, JUNO will lead the precision of about half of neutrino oscillation parameters.

However, JUNO has been designed to yield a unique MO sensitivity via vacuum oscillation upon the spectral distortion $3\nu$ analysis formulated in terms of $\delta m^2_{21}$ and $\Delta m^2_{32}$ (or $\Delta m^2_{31}$). JUNO’s MO sensitivity relies on a challenging experimental articulation for the accurate control of the spectral shape-related systematics arising from energy resolution, energy scale control (nonlinearities being the most important), and even the reactor reference spectra to be measured independently by the TAO experiment^47^. The nominal intrinsic MO sensitivity is $\sim 3\sigma$ ($\Delta \chi ^2$ $\approx 9$) upon 6 years of data taking. All JUNO inputs to this paper follow the JUNO collaboration prescription^24^, including $\Delta m^2_{32}$. Hence, JUNO alone is unable to resolve MO with high level of confidence ($\Delta \chi ^2$ $\ge 25$) in a reasonable time. In our simplified approach, we shall characterise JUNO by a simple $\Delta \chi ^2$ = $9\pm 1$. The uncertainty aims to illustrate possible minor variations in the final sensitivity due to the experimental challenges behind or improvements in the analysis.

### Mass ordering resolution power in LB$\nu$B-II

In all LB$\nu$B experiments, the intrinsic MO sensitivity arises via the *appearance channel* (AC), from the transitions $\nu _\mu \rightarrow \nu _e$ and ${\bar{\nu }}_\mu \rightarrow {\bar{\nu }}_e$; also sensitive to $\delta _{\tiny \text {CP}}$. MO manifests as an effective *fake* CPV effect or bias. This effect causes the oscillation probabilities to be different for neutrino and anti-neutrinos even under CP-conserving solutions. It is not trivial to disentangle the genuine ($\delta _{\tiny \text {CP}}$) and the faked CPV terms. Two main strategies exist, based on the fake component, which is to be either (a) minimised (i.e. shorter baseline, like T2K, 295 km) enabling to measure mainly $\delta _{\tiny \text {CP}}$ or (b) maximised (i.e. longer baseline), so that matter effects are strong enough to disentangle them from the $\delta _{\tiny \text {CP}}$, and both can be measured simultaneously exploiting spectral information from the second oscillation maximum. The latter implies baselines $> 1000\,$km, best represented by DUNE ($1300\,$km). NOvA’s baseline ($810\,$km) remains a little too short for a full disentangling ability. Still, NOvA remains the most important LB$\nu$B to date with sizeable intrinsic MO sensitivity due to its relatively large matter effects as compared to T2K.

**Figure 2:**
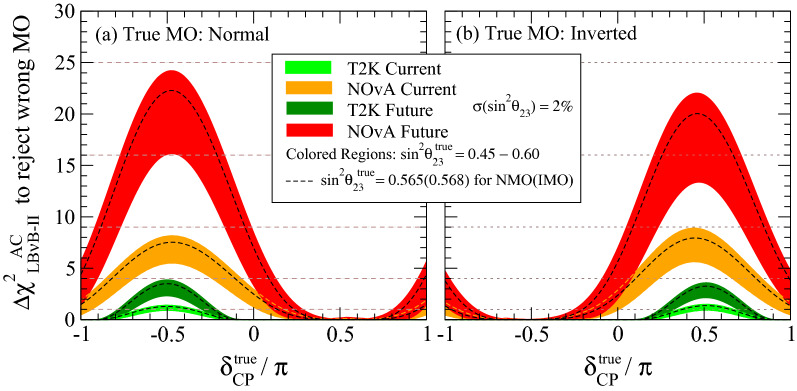
LB$\nu$B-II mass ordering sensitivity. The Mass Ordering (MO) sensitivity of LB$\nu$B-II experiments via the appearance channel (AC), constrained to a range of $\theta _{23}$, is shown as a function of the “true” value of $\delta _{\tiny \text {CP}}$. The bands represent the cases where the “true” value of $\sin ^2\theta _{23}$ lies within the interval [0.45, 0.60] with a relative experimental uncertainty of 2%. The $\sin ^2\theta _{23} = 0.60$ (0.45) gives the maximum (minimum) sensitivity for a given value of $\delta _{\tiny \text {CP}}$. The black dashed curves indicate the NuFit5.0 best fitted $\sin ^2\theta _{23}$ value. The NMO and IMO sensitivities are illustrated respectively in the (**a**) and (**b**) panels. The sensitivity arises from the fake CPV effect due to matter effects, proportional to the baseline (*L*). The strong dependence on $\delta _{\tiny \text {CP}}$ is due to the unavoidable degeneracy between NMO and IMO, thus causing the sensitivity to swig by 100%. T2K, now (light green) and future (dark green), exhibits minimal intrinsic sensitivity due to its shorter baseline ($L_{\tiny \text {T2K}}= 295$  km). Instead, NOvA, now (orange) and future (red), hold leading order MO information due to its larger baseline ($L_{\tiny \text {NOvA}}= 810$ km). The future full exposure for T2K and NOvA implies $\sim 3$ times more statistics relative to today. These curves are referred to as $\Delta {\chi ^2}_{\text {LB}\nu \text {B}}^\text { AC}$ and were derived from data as detailed in Appendix A.

Figure 2 shows the current and future intrinsic MO sensitivities of LB$\nu$B-II experiments, including their explicit $\theta _{23}$ and $\delta _{\tiny \text {CP}}$ dependencies. The obtained MO sensitivities were computed using a simplified strategy where the AC was treated as *rate-only* (i.e., one-bin counting) analysis, thus neglecting any shape-driven sensitivity gain. This approximation is remarkably accurate for off-axis beams (narrow spectrum), especially in the low statistics limit, where the impact of systematics remains small (here neglected). The background subtraction was accounted for and tuned to the latest experiments’ data. To corroborate our estimate’s accuracy, we reproduced the LB$\nu$B-II latest results^20^, as detailed in Appendix A.

While NOvA AC holds significant intrinsic MO information, it is unlikely to resolve ($\Delta \chi ^2$ $\ge$25) alone. This outcome is similar to that of JUNO. Of course, the natural question may be whether their combination could yield the full resolution. Unfortunately, as it will be shown, this is unlikely but not far. Therefore, in the following, we shall consider their combined potential, along with T2K, to provide the extra missing push. This may be somewhat counter-intuitive since T2K has just been shown to hold minimal intrinsic MO sensitivity, i.e., $\le$4 units of $\Delta \chi ^2$. Indeed, T2K, once combined, has an alternative path to enhance the overall sensitivity, which is to be described next.

### Synergetic mass ordering resolution power

A remarkable synergy exists between JUNO and LB$\nu$B experiments thanks to their complementarity^24,43–45,50,51^. In this case, we shall explore the contribution via the LB$\nu$B’s *disappearance channel* (DC), i.e., the transitions $\nu _\mu \rightarrow \nu _\mu$ and ${\bar{\nu }}_\mu \rightarrow {\bar{\nu }}_\mu$. This might appear counter-intuitive, since DC is practically blinded (i.e. variations $<1\%$) to MO, as shown in Appendix-B.

Instead, the LB$\nu$B DC provides a precise complementary measurement of $\Delta m^2_{32}$. This information unlocks a mechanism, described below, enabling the intrinsic MO sensitivity of JUNO to be enhanced by the external $\Delta m^2_{32}$ information. This highly non-trivial synergy may yield a MO leading order role but introduces new dependences, also explored below.

Both JUNO and LB$\nu$B analyse data in the 3$\nu$ framework to directly provide $\Delta m^2_{32}$ (or $\Delta m^2_{31}$) as output. The 2$\nu$ approximation leads to effective observables, such as $\Delta m^2_{\mu \mu }$ and $\Delta m^2_{ee}$^43^ detailed in Appendix-C. A CP-driven ambiguity limits the LB$\nu$B DC information precision on the $\Delta m^2_{32}$ measurement if LB$\nu$B AC measurements are not taken into account. The role of this ambiguity is small, but not entirely negligible and will be detailed below. The dominant LB$\nu$B-II’s precision is today $\sim 2.9\%$ per experiment^52,53^. The combined LB$\nu$B-II global precision on $\Delta m^2_{32}$ is already $\sim 1.4\%$^21^. Further improvement below 1.0% appears possible within the LB$\nu$B-II era when integrating the full luminosities^53,54^. An average precision of $\sim 0.5\%$ is reachable only upon the next LB$\nu$B-III generation. Instead, JUNO precision on $\Delta m^2_{32}$ is expected to be well within the sub-percent ($<0.5\%$) level^24,55^.

**Figure 3:**
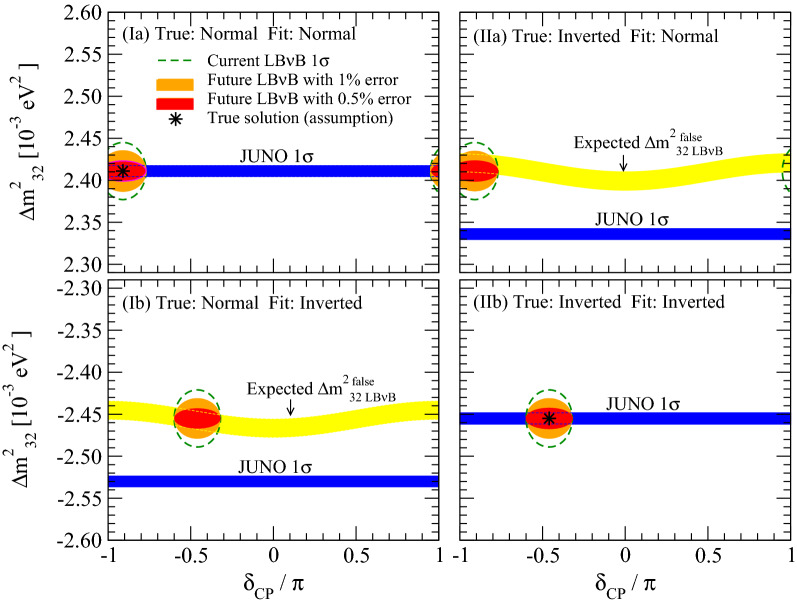
Origin of MO Boosting by LB$\nu$B for JUNO. Semi-quantitative and schematic illustration of the LB$\nu$B JUNO MO resolution synergy is shown for the cases where the true MO is normal (left panels) or inverted (right panels). For each case, the true values of $\Delta {m^2_{32}}$ are assumed to coincide with the NuFit5.0 best fitted values indicated by the black asterisk symbols. For each assumed true value of $\Delta {m^2_{32}}$, possible range of the false values of $\Delta {m^2_{32}}$ to be determined from LB$\nu$B DC is indicated by the yellow color bands where their width reflects the ambiguity due to the CP phase (see Appendix C). The approximate current 1$\sigma$ allowed ranges of ($\delta _{\tiny \text {CP}},\,\Delta {m^2_{32}}$) from NuFit5.0 are indicated by the dashed green curve whereas the future projections assuming the current central values with 1% (0.5%) uncertainty of $\Delta m^2_{32}$ are indicated by filled orange (red) color. Expected 1$\sigma$ ranges of $\Delta m^2_{32}$ from JUNO alone are indicated by the blue color bands though the ones in the wrong MO region would be disfavored at $\sim 3\sigma$ confidence level (CL) by JUNO itself. When the MO which is assumed in the fit coincides with the true one, allowed region of $\Delta {m^2_{32}}$ by LB$\nu$B overlaps with the one to be determined by JUNO as shown in the panels I(a) and II(b). On the other hand, when the assumed (true) MO and fitted one do not coincide, the expected (false) values of $\Delta {m^2_{32}}$ by LB$\nu$B and JUNO do not agree, as shown in the panels I(b) and II(a), disfavouring these cases, which is the origin of what we call the boosting effect in this paper.

The essence of the synergy is described here. Upon 3$\nu$ analysis, both JUNO and LB$\nu$B experiments obtain two different values for $\Delta m^2_{32}$ depending on the assumed MO. Since there is only one *true* solution, NMO, or IMO, the other solution is thus *false*. The standalone ability to distinguish between those two solutions is the *intrinsic* MO resolution power of each experiment. The critical observation is that the general relation between the true-false solutions is different for reactors and LB$\nu$B experiments, as *semi-quantitatively* illustrated in Fig. 3. For a given true $\Delta m^2_{32}$, its false value, referred to as $\Delta {m^2_{32}}^\text {false}$, as detailed in Appendix C. This implies that both JUNO and LB$\nu$B based experiments generally have 2 solutions corresponding to NMO and IMO, illustrated in Fig. 3 by the region delimited by the dashed green ellipses for the current LB$\nu$B  data and blue bands for JUNO. The yellow bands indicate the possible range of false $\Delta m^2_{32}$ values expected from LB$\nu$B, including a $\delta _{\tiny \text {CP}}$ dependence, if the current best fit $\Delta m^2_{32}$ is turned out to be true.

All experiments must agree on the unique true $\Delta m^2_{32}$ solution. Consequently, the corresponding JUNO ($\Delta {m^2_{32}}^\text {false}_{\tiny \text { JUNO}}$) and LB$\nu$B ($\Delta {m^2_{32}}^\text {false}_{\tiny {\text { LB}\nu \text {B}}}$) false solutions will differ if the overall $\Delta m^2_{32}$ precision allows their relative resolution. The ability to distinguish (or separate) the false solutions, or *mismatch* of 2 false solutions, seen in the panels (Ib) and (IIa) in Fig. 3, can be exploited as an extra dedicated discriminator expressed by the term:

1 $$\begin{aligned} \Delta \chi ^2_{\tiny \text {BOOST}} \sim \left( \frac{ \Delta {m^2_{32}}^{\tiny \text {false}}_{\tiny \text { JUNO}}- \Delta {m^2_{32}}^{\tiny \text {false}}_{\tiny {\text { LB}\nu \text {B}}}}{\sigma (\Delta m^2_{32})_{\tiny {\text {LB}\nu \text {B}}}} \right) ^2. \end{aligned}$$

This $\Delta \chi ^2_{\tiny \text {BOOST}}$ term characterises the rejection of the false solutions (either NMO or IMO) through an hyperbolic dependence on the overall $\Delta m^2_{32}$ precision. The derived MO sensitivity enhancement may be so substantial that it can be regarded and as a potential *boost* effect in the MO sensitivity.

**Figure 4:**
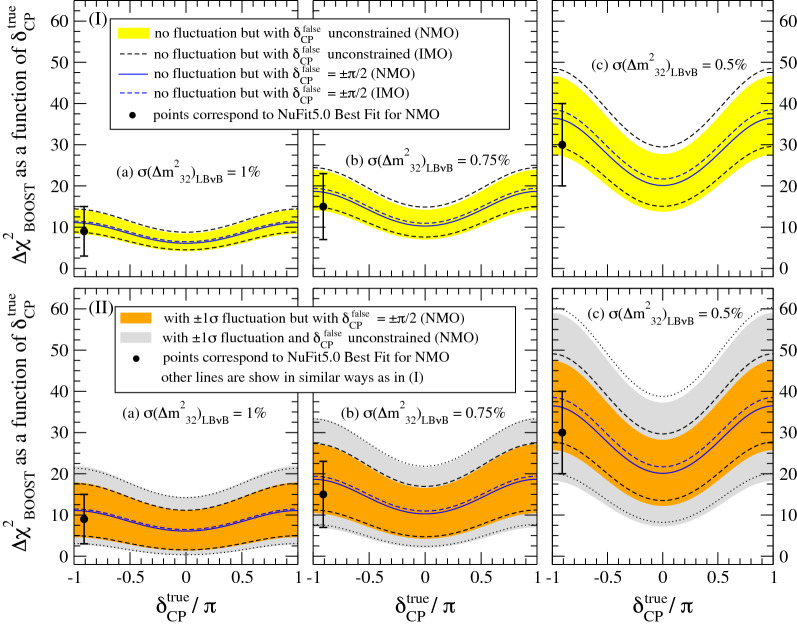
JUNO and LB$\nu$B mass ordering synergy dependences. The isolated synergy boosting term obtained from the combining JUNO and LB$\nu$B experiments is represented by $\Delta \chi ^2_{\tiny \text {BOOST}}$, as approximately shown in Eq. (1), see Appendix-C for details. $\Delta \chi ^2_{\tiny \text {BOOST}}$ depends on the true value of $\delta _{\tiny \text {CP}}$ and $\Delta m^2_{32}$ precision, where uncertainties are considered: 1.0% (a), 0.75% (b) and 0.5% (c). The $\Delta \chi ^2_{\tiny \text {BOOST}}$ term is almost identical for both NMO and IMO solutions. Two specific effects lead the uncertainty in the a priori prediction on $\Delta \chi ^2_{\tiny \text {BOOST}}$. (I) illustrates only the ambiguity of the CP phase (yellow band) impact whereas (II) shows only the impact of the $\pm 1\sigma$ fluctuations of $\Delta m^2_{32}$, as measured by LB$\nu$B (orange band). The JUNO uncertainty on $\Delta m^2_{32}$ is considered to be less than $0.5\%$. The grey bands in (II) show when both effects are taken into account simultaneously. The mean value of the $\Delta \chi ^2_{\tiny \text {BOOST}}$ term increases strongly with the precision on $\Delta m^2_{32}$. The uncertainties from CP phase ambiguity and fluctuation could deteriorate much of the a priori gain on the prospected sensitivities. $\Delta m^2_{32}$ fluctuations dominate, while the $\delta _{\tiny \text {CP}}$ ambiguity is only noticeable for the best $\Delta m^2_{32}$ precision. The use of NuFit5.0 data (black point) eliminates the impact of the $\delta _{\tiny \text {CP}}$ prediction ambiguity while the impact of $\Delta m^2_{32}$ remains as fluctuations cannot be predicted a priori. Today’s favoured $\delta _{\tiny \text {CP}}$ maximises the sensitivity gain via the $\Delta \chi ^2_{\tiny \text {BOOST}}$ term. When quoting sensitivities, we shall consider the lowest bound as the most conservative case.

**Figure 5:**
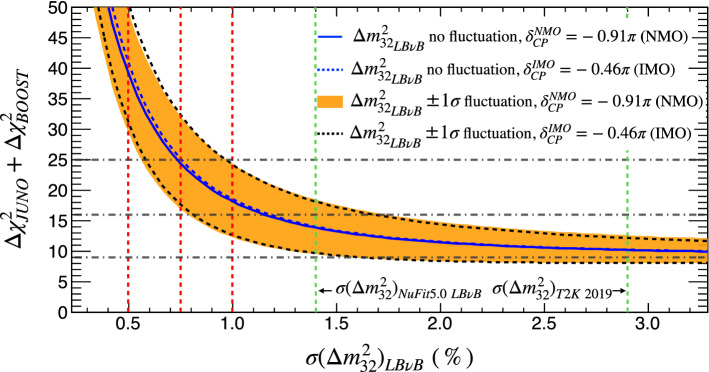
JUNO mass ordering sensitivity boosting. A significant increase of JUNO intrinsic sensitivity ($\Delta \chi ^2_{\tiny \text {JUNO}} \approx 9$) is possible exploiting the LB$\nu$B’s disappearance (DC) characterised by $\Delta \chi ^2_{\tiny \text {BOOST}}$ depending strongly on the uncertainty of $\Delta m^2_{32}$. Today’s NuFit5.0 average LB$\nu$B-II’s precision on $\Delta m^2_{32}$ is $\sim 1.4\%$. A rather humble 1.0% precision is possible, consistent with doubling the statistics if systematics allowed. Since NOvA and T2K are expected to increase their exposures by about factors of $\sim 3$ before the shutdown, sub-percent precision may also be within reach. While the ultimate precision is unknown, we shall consider a $\ge 0.75\%$ precision to illustrate this possibility. So, JUNO alone (intrinsic + boosting) could yield a $\ge 4\sigma$ (i.e., $\Delta \chi ^2$ $\ge$16) MO sensitivity, at $\ge$84% probability, within the LB$\nu$B-II era. A 5$\sigma$ potential may not be impossible, depending on fluctuations. Similarly, JUNO may further increase in significance to resolve ($\ge 5\sigma$ or $\Delta \chi ^2$ $\ge 25$) a pure vacuum oscillations MO measurement in combination with the LB$\nu$B-III’s $\Delta m^2_{32}$ information.

**Figure 6:**
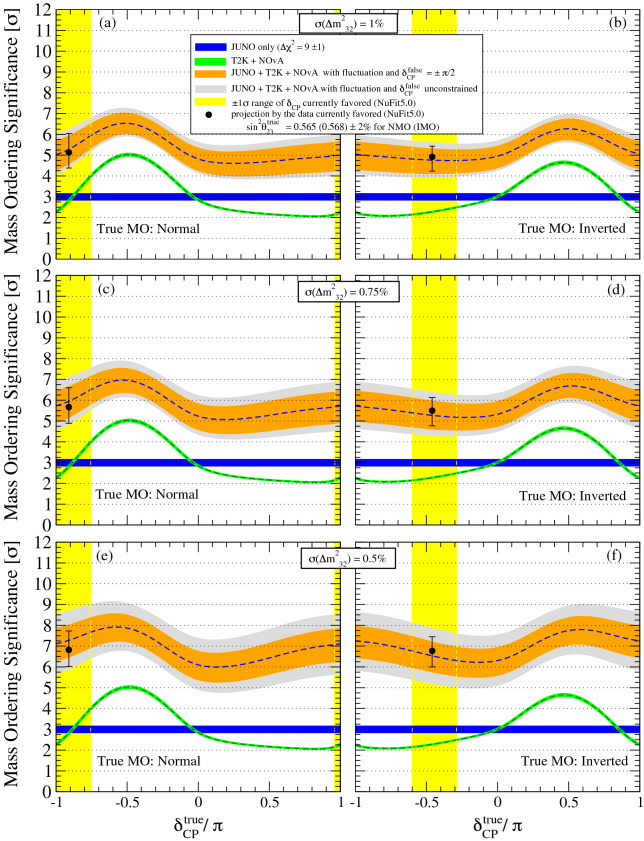
The combined mass ordering sensitivity. The combination of the MO sensitive of JUNO and LB$\nu$B-II is illustrated for six difference configurations: NMO (left), IMO (right) considering the LB$\nu$B uncertainty on $\Delta m^2_{32}$ to 1.0% (top), 0.75% (middle) and 0.5% (bottom). The NuFit5.0 favoured value is set for $\sin ^2 \theta _{23}$ with an assumed 2% experimental uncertainty. The intrinsic MO sensitivities are shown for JUNO (blue) and the combined LB$\nu$B-II (green), the latter largely dominated by NOvA. The JUNO sensitivity boosts when exploiting the LB$\nu$B’s $\Delta m^2_{32}$ additional information via the $\Delta \chi ^2_{\tiny \text {BOOST}}$ term, described in Fig. 4 but not shown here for illustration simplicity. The orange and grey bands illustrate the presence of the boosting term prediction effects, respectively, the $\pm 1\sigma$ fluctuation of $\Delta m^2_{32}$ and the $\delta _{\tiny \text {CP}}$ ambiguity in addition. T2K impacts mainly via the precision of $\Delta m^2_{32}$ and the measurement of $\delta _{\tiny \text {CP}}$. The combined sensitivity suggests a mean (dashed blue line) $\ge 4\sigma$ significance for any value of $\delta _{\tiny \text {CP}}$ even for the most conservative $\sigma (\Delta m^2_{32})=$1%. However, a robust $\ge 5.0\sigma$ significance at 84% probability (i.e. including fluctuations) seems possible, if the currently preferred value of $\delta _{\tiny \text {CP}}$ and NMO remain favoured by data, as indicated by the yellow band and black point (best fit). Further improvement in the precision of $\Delta m^2_{32}$ translates into a better MO resolution potential.

**Figure 7:**
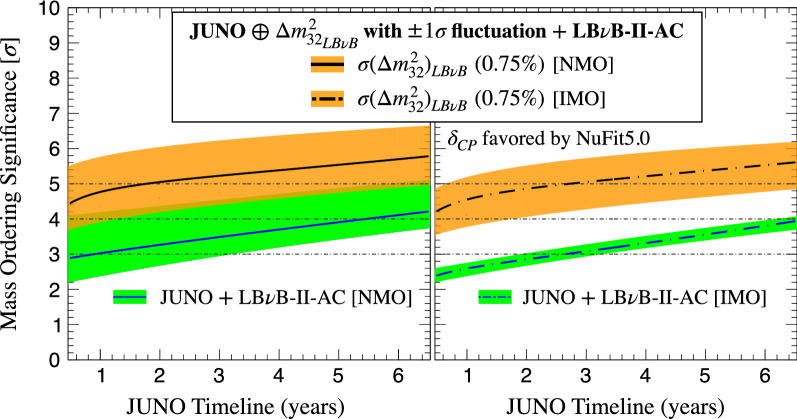
Mass ordering sensitivity and possible resolution timeline. Since all the NOvA and T2K data are expected to be accumulated by $\sim$2024^57^ and $\sim$2026^52^, the combined sensitivity follows JUNO data availability. JUNO is expected to start in 2023, reaching its statistically dominated nominal MO sensitivity (9 units of $\Delta \chi ^2$) within $\sim$6 years. We illustrate the NMO (plot on the left) and IMO (plot on the right) scenarios. The sensitivity evolution depends mainly on JUNO once boosted, where 0.75% $\Delta m_{32}^2$ uncertainty (black line) is considered. The effect of $\Delta m^2_{32}$ fluctuations is indicated (orange bands), including that of the variance due to the data favoured region for $\delta _{\tiny \text {CP}}$ (green band). The larger band of the NMO is caused by contribution of the LB$\nu$B-II experiments, whose contribution is rather negligible for opposite IMO. Since JUNO boosted dominates, the sensitivities are almost independent of NMO and IMO solutions (left), this also demonstrating the humble overall impact of the AC channel (NOvA mainly) of the LB$\nu$B-II experiments upon combination. The mean significance is expected to reach the $\sim 5\sigma$ level, including fluctuations and degeneracies (i.e., $\ge$84% probability) for both MO solutions, where the precision on $\Delta m^2_{32}$ is the leading order term. In fact, a 5$\sigma$ measurement may be possible, at 50% probability, within 3 years of JUNO data taking start once combined. In the end, JUNO data may prescind entirely from the LB$\nu$B’s AC information (minor impact), thus enabling a fully resolved pure vacuum oscillation MO measurement.

The JUNO-LB$\nu$B boosting synergy exhibits four main features as illustrated in Fig. 4:

- *Major increase (boost) potential of the combined MO sensitivity.* This is realised by the new pull term, shown in Eq. (1) and illustrated in Fig. 4, which is to be added to the intrinsic MO discrimination $\Delta \chi ^2$ terms per experiment as it will be described later on in Figs. 5, 6, 7.
- *Dependence on the precision of* $\Delta m^2_{32}$**.** Again, this is described explicitly in Eq. (1). The leading order effect is the uncertainty on $\Delta m^2_{32}$. This typically referred to as $\sigma (\Delta m^2_{32})_{\text {LB}\nu \text {B}}$ as this largely dominates due to its poorer precision as compared to that obtained by JUNO ($\le$ 0.5%) even within about a year of data-taking. Three cases are explored in this work, (a) 1.0% (i.e. close to today’s precision), (b) 0.75% and (c) 0.5% (ultimate precision). Figure 4 exhibits a strong dependence, telling us the importance of reducing the uncertainties of $\Delta m^2_{32}$ from LB$\nu$B to increase the MO sensitivity. This is why T2K can have an active and important role to improve the overall MO sensitivity.
- *Impact of fluctuations.* In order to be accurately predictive, it is important to evaluate the impact of the unavoidable fluctuations due to the today’s data uncertainties on $\Delta m^2_{32}$ as well as on the $\delta _{\tiny \text {CP}}$ ambiguity (see below description). All these effects are quantified and explained in Fig. 4 by the orange bands, thus representing the $\pm 1\sigma$ data fluctuations of $\Delta m^2_{32}$ from LB$\nu$B can significantly impact the boosted MO sensitivity.
- *δ*_*CP* _*Ambiguity dependence*. The main consequence is to limit the predictability of $\Delta \chi ^2_{\tiny \text {BOOST}}$, even if the assumed true value of the CP phase is fixed or limited to very narrow range. Its effect is less negligible as the LB$\nu$B precision on $\Delta m^2_{32}$ improves ($\le$0.5%), as shown by the yellow bands in (I) and by the gray band in (II) of Fig. 4. However, by considering the $\Delta m^2_{32}$ determined by the global fit like NuFit5.0, we can reduce this ambiguity as the best fitted $\Delta m^2_{32}$ values for NMO and IMO also reflect the most likely values of $\delta _{\tiny \text {CP}}$ maximising our predictions’ accuracy to the most probable parameter-space, as favoured by the latest world neutrino data [despite that $\Delta \chi ^2_\text {boost}$ defined by Eqs. (15) and (16) in Appendix-C does not depend explicitly on the CP phase, we are implicitly using the CP phase information since the best fitted $\Delta m^2_{32}$ coming from the global analysis carry the informtion on $\delta _{{\tiny \text {CP}}}$ through the LB$\nu$BAC data used in the global analysis].

In brief, when combining JUNO and the LB$\nu$B experiments, the overall sensitivity works as if JUNO’s intrinsic sensitivity gets boosted, via the external $\Delta m^2_{32}$ information. This is further illustrated and quantified in Fig. 5, as a function of the precision on $\Delta m^2_{32}$ despite the sizeable impact of fluctuations. The LB$\nu$B intrinsic AC contribution will be added and shown in the next section. It is also demonstrated that the DC information of the LB$\nu$B’s, via the boosting, play a significant role in the overall MO sensitivity. However, this improvement cannot manifest without JUNO – and vice versa. For an average precision on $\Delta m^2_{32}$ below 1.0%, even with fluctuations, the boosting effect can be already considerable. A $\Delta m^2_{32}$ precision as good as $> 0.75\%$ may be accessible by LB$\nu$B-II while the LB$\nu$B-III generation is expected to go up to $\le 0.5\%$ level.

Since the exploited DC information is practically blinded to matter effects [the $\Delta m^2_{32}$ measurement of depends slightly on $\delta _{\tiny \text {CP}}$, obtained via the AC information, itself sensitive to matter effects], the boosting synergy effect remains dominated by JUNO’s vacuum oscillations nature. For this reason, the sensitivity performance is almost identical for both NMO and IMO solutions, in contrast to the sensitivities obtained from solely matter effects, as shown in Fig. 2. This effect is especially noticeable in the case of atmospheric data. The case of T2K is particularly illustrative, as its impact on MO resolution is essentially only via the boosting term mainly, given its small intrinsic MO information obtained by AC data. This combined MO sensitivity boost between JUNO and LB$\nu$B (or atmospherics) is likely one of the most elegant and powerful examples so far seen in neutrino oscillations, and it is expected to play a significant role for JUNO to yield a leading impact on the MO quest, as described next. In fact, the JUNO collaboration has already considered this effect when claiming its possible median MO sensitivity to be 4$\sigma$ potential^24,44^. However, JUNO prediction does not account for the $\Delta m^2_{32}$ fluctuations. This work adds the impact of $\Delta m^2_{32}$ fluctuations and $\delta _{\tiny \text {CP}}$ ambiguity on the MO discovery potential of JUNO upon boosting. Our results are however consistent if used the same assumptions, as described in Appendix D.

## Simplified combination rationale

The combined MO sensitive of JUNO together with LB$\nu$B-II experiments (NOvA and T2K) can be obtained from the independent additive of each $\Delta \chi ^2$. Two contributions are expected: a) the LB$\nu$B-II’s AC, referred to as $\Delta \chi ^2$(LB$\nu$B-AC) and b) the combined JUNO and LB$\nu$B-II’s DC, referred to as $\Delta \chi ^2$(JUNO$\oplus$LB$\nu$B-DC). All terms were described in the previous sections [we use in this work the terminologies, AC (appearance channel) and DC (disappearance channel) for simplicity. This does not mean that the relevant information is coming only from AC or DC, but that $\Delta$ $\chi ^2$(LB$\nu$B-AC) comes dominantly from LB$\nu$B AC whereas $\Delta$ $\chi ^2$(JUNO$\oplus$LB$\nu$B-DC) comes dominantly from JUNO + LB$\nu$B DC]. Hence the combination can be represented as $\Delta \chi ^2$ = $\Delta \chi ^2$(JUNO$\oplus$LB$\nu$B-DC) + $\Delta \chi ^2$(LB$\nu$B-AC), illustrated in Fig. 6, where the orange and grey bands represent, respectively, the effects of the $\Delta m^2_{32}$ fluctuations and the CP-phase ambiguity. Figure 6 quantifies the MO sensitivity in terms of significance (i.e., numbers of $\sigma$’s) obtained as $\sqrt{\Delta \chi ^2}$ quantified in all previous plots. Again, both NMO and IMO solutions are considered for 3 different cases for the LB$\nu$B uncertainty on $\Delta m^2_{32}$.

The $\Delta \chi ^2$(LB$\nu$B-II-AC) Term:
this is the intrinsic MO combined information, largely dominated by NOvA’s AC, as described in Fig. 2. The impact of T2K ($\le 2\sigma$) is minimal, but on the verge of resolving MO for the first time, T2K may still help here. As expected, this $\Delta \chi ^2$ depends on $\theta _{23}$ and strongly on $\delta _{\tiny \text {CP}}$. This is shown in Fig. 6 by the light green band. We note that when T2K and NOvA are combined, there is $\sim 2\sigma$ significance enhancement in the positive (negative) range of $\delta _{\tiny \text {CP}}$ for NMO (IMO) which is not naively expected from Fig. 2. This extra gain of sensitivity for the T2K and NOvA combined case comes from the difference of the matter effects on these experiments, and can be seen, e.g., in Figure 21 of Ref.^56^. The complexities of possible correlations and systematics handling of a hypothetical NOvA and T2K combination are disregarded in our study, but they are integrated within the combination of the LB$\nu$B-II term, now obtained from NuFit5.0. The full NOvA data is expected to be available by 2024^57^, while T2K will run until 2026^52^, upon the beam upgrades (T2K-II) aiming for HK.
The $\Delta \chi ^2$(JUNO$\oplus$LB$\nu$B-DC) Term:
this term can be regarded itself as composed of two contributions. The first part is the JUNO intrinsic information, i.e., $\Delta \chi ^2$ = $9\pm 1$ units after 6 years of data-taking. This contribution is independent of $\theta _{23}$ and $\delta _{\tiny \text {CP}}$, as shown in Fig. 6, represented by the blue band. The second part is the JUNO boosting term, shown explicitly in Fig. 4, including its generic dependencies, such as the true value of $\delta _{\tiny \text {CP}}$. This term exhibits strong modulation with $\delta _{\tiny \text {CP}}$ and uncertainty of $\Delta m^2_{32}$, as illustrated in Figs. 4 and 5. The $\Delta \chi ^2$(JUNO$\oplus$LB$\nu$B-DC) term strongly shapes the combined $\Delta \chi ^2$ curves (orange). Indeed, this term causes the leading variation across Fig. 6 for the different cases of the uncertainty of $\Delta m^2_{32}$: (a) 1.0% (top), reachable by LB$\nu$B-II^53,54^, (b) 0.75% (middle), maybe reachable (i.e. optimistic) by LB$\nu$B-II and (c) 0.5% (bottom), which is only reachable by the LB$\nu$B-III generation^31,32^.

The combination of the JUNO, AC, and DC inputs from LB$\nu$B-II experiments appears on the verge of achieving the first MO resolved measurement with a sizeable probability. The combination’s ultimate significance is likely to mainly depend on the final uncertainty on $\Delta m^2_{32}$ obtained by LB$\nu$B experiments. The discussion of the results and implications, including limitations, is addressed in the next section.

## Implications and discussion

Possible implications arising from the main results summarised in Fig. 6 deserved some extra elaboration and discussion for a more accurate contextualisation, including a possible timeline and highlight the limitations associated with our simplified approach. These are the main considerations:

1. *MO global data trend:* Today’s reasonably high significance, not far from the level to be reached by intrinsic sensitivities of JUNO or NOvA, is obtained by the most recent global analysis^21^ which favours NMO up to $2.7\sigma$. However, this significance lowers to $1.6\sigma$ without SK atmospherics data, thus proving their crucial value to the global MO knowledge today. The remaining aggregated sensitivity integrates over all other experiments. However, the global data preference is somewhat fragile, still varying between NMO and IMO solutions^17,21,58^.
  The reason behind this is actually the corroborating manifestation of the alluded complementarity between LB$\nu$B-II and *reactors* [before JUNO starts, the reactor experiments stand for Daya Bay, Double Chooz, and RENO, whose lower precision on $\Delta m^2_{32}$ is $\sim$2%] experiments. Indeed, while the current LB$\nu$B data alone favour IMO, the match in $\Delta m^2_{32}$ measurements by LB$\nu$B and reactors tend to favour the case of NMO, which is this overall solution obtained upon combination. Hence, the MO solution currently flips due to the reactor-LB$\nu$B data interplay, despite the sizeable $\Delta m^2_{32}$ uncertainty fluctuations as compared to the aforementioned scenario where JUNO will be on, indicating it’s crucial contribution. This effect, expected since^43^, is at the heart of the described boosting mechanism and has started manifesting earlier on. This can be regarded as the first data-driven manifestation of the aforementioned $\Delta \chi ^2_{\tiny \text {BOOST}}$ effect.
2. *Atmospherics extra information:* We did not account for atmospheric neutrino input, such as the running SK and IceCube experiments. They are expected to add valuable $\Delta \chi ^2$though susceptible to the aforementioned $\theta _{23}$ (mainly) and $\delta _{\tiny \text {CP}}$ dependences. This contribution is more complex to replicate with accuracy due to the vast *E*/*L* phase-space; hence we disregarded it in our simplified analysis. Its importance has long been proved by SK dominance of much of today’s MO information. So, all our conclusions can only be enhanced by adding the missing atmospheric contribution. Future ORCA and PINGU have the potential to yield extra MO information^45^, while their combinations with JUNO data is actively studied^59,60^ to yield full MO resolution.
3. *Inter-experiment full combination:* A complete strategy of data-driven combination between JUNO and LB$\nu$B-II experiments will be beneficial in the future [during the final readiness of our work, one such a combination was reported^61^ using a different treatment (excluding fluctuations). While their qualitative conclusions are consistent with our studies, there may still be numerical differences left to be understood]. Ideally, this may be an official inter-collaboration effort to carefully scrutinise the possible impact of systematics and correlations, involving both experimental and theoretical physicists in such studies (see e.g.^51^). We do not foresee a significant change in our findings by a more complex study, including the highlighted MO discovery potential due to today’s data and knowledge limitations.
  Our approach did not merely demonstrate the numerical yield of the combination between JUNO and LB$\nu$B, but our goal was also to illustrate and characterise the different synergies manifesting therein. Our study focuses on the breakdown of all the relevant contributions in the specific and isolated cases of the MO sensitivity combination of the leading experiments. The impact of the $\Delta \chi ^2_{\tiny \text {BOOST}}$ was isolated, while its effect is otherwise transparently accounted for by any complete 3$\nu$ $\chi ^2$ formulation, such as done by NuFit5.0 or other similar analyses. Last, our study was tuned to the latest data to maximise the accuracy of predictability, which is expected to be order $\sim 0.5\sigma$ around the 5$\sigma$ range.
4. *Hypothetical MO resolution timeline:* One of the main observations upon this study is that the MO could be fully resolved, maybe even comfortably, by the JUNO, NOvA and T2K combination. The NMO solution discovery potential, considering today’s favoured $\delta _{\tiny \text {CP}}$, has a probability of $\ge 50\%$ ($\ge 84\%$) for a $\Delta m^2_{32}$ precision of up to 1.0% (0.75%). In the harder IMO, the sensitivity may reach a mean of $\sim 5\sigma$ potential only if the $\Delta m^2_{32}$ uncertainty was as good as $\sim 0.75\%$. Within a similar time scale, the atmospheric data is expected to add up to enable a full $5\sigma$ resolution for both solutions. If correct, this is likely to become the first fully resolved MO measurement and it is expected to be tightly linked to the JUNO data timeline, as described in Fig. 7, which sets the timeline to be between 2026–2028.
  Such a combined MO measurement can be regarded as a “hybrid” between vacuum (JUNO) and matter driven (mainly NOvA) oscillations. In this context, JUNO and NOvA are, unsurprisingly, the leading experiments. Despite holding little intrinsic MO sensitivity, T2K plays a key role by simultaneously a) boosting JUNO via its precise measurement of $\Delta m^2_{32}$ (similar to NOvA) and b) aiding NOvA by reducing the possible $\delta _{\tiny \text {CP}}$ ambiguity phase-space. The Appearance Channel channel synergy between T2K and NOvA is expected to have very little impact.
  This combined measurement relies on an impeccable $3\nu$ data model consistency across all experiments. Possible inconsistencies may diminish the combined sensitivity. Since our estimate has accounted for fluctuations (typically, up to $\sim 84\%$ probability), those inconsistencies should amount to $\ge 2\sigma$ effects for them to matter. Those inconsistencies may, however, be the first manifestation of new physics^62,63^. Hence, this inter-experiment combination has another relevant role: to exploit the ideal MO binary parameter space solution to test for inconsistencies that may point to discoveries beyond today’s standard picture. The additional atmospherics data mentioned above, are expected to reinforce both the significance boost and the model consistency scrutiny just highlighted.
5. *Readiness for LB*$\nu$*B-III:* in the absence of any robust model-independent for MO prediction by theory and given its unique binary MO outcome, the articulation of at least two well resolved measurements appears critical for the sake of the experimental redundancy and consistency test across the field. In the light of DUNE’s unrivalled MO resolution power, the articulation of another robust MO measurement may be considered as a priority to make the most of DUNE’s insight.
6. *Vacuum versus matter measurements:* since matter effects drive all experiments but JUNO, articulating a competitive and fully resolved measurement via only vacuum oscillations has been an unsolved challenge to date. Indeed, boosting JUNO sensitivity alone, as described in Figs. 4 and 5, up to $\ge 5\sigma$ remains likely impractical in the context of LB$\nu$B-II, modulo fluctuations. However, this possibility is a priori feasible in combination with the LB$\nu$B-III improved precision, as shown in Fig. 7 and more detailed Fig. 8. The significant potential improvement in the $\Delta m^2_{32}$ precision, up to order 0.5%^31,32^ may prove crucial. Furthermore, the comparison between two fully resolved MO measurements, one using only *matter effects* and one exploiting pure *vacuum oscillations*, is foreseen to be one of the most insightful MO coherence tests. So, the ultimate MO measurements comparison may be the DUNE’s AC alone (even after a few years of data taking) versus a full statistics JUNO boosted by the DC of HK and DUNE improving the $\Delta m^2_{32}$ precision. This comparison is expected to maximise the depth of the MO-based scrutiny by their stark differences in terms of mechanisms, implying dependencies, correlations, etc. The potential for a breakthrough or even discovery, exists, should a significant discrepancy manifest here. The expected improvement in the knowledge of $\delta _{\tiny \text {CP}}$ by LB$\nu$B-III experiments will also play a role in facilitating this opportunity.

**Figure 8:**
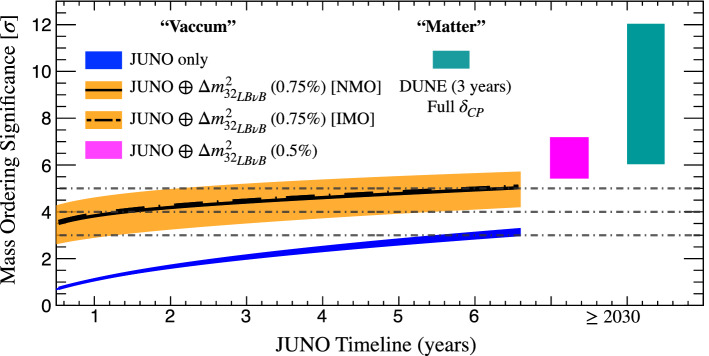
Different measurements of mass ordering. As illustrated in Fig. 7, the LB$\nu$B-II generation may provide major boosting of the JUNO sensitivity upon the boosting caused by $\Delta m^2_{32}$ precision, which is expected to be at best $\ge 0.75\%$. The boosting effect is again illustrated as the difference between the JUNO alone (blue) and JUNO boosted (orange) curves. However, once the LB$\nu$B-III generation accelerator experiments start, we expect the $\Delta m^2_{32}$ precision to be further enhanced up to $\sim 0.5\%$ by both DUNE and HK. At this moment, the JUNO data may only exploit this $\Delta m^2_{32}$ precision to ensure a fully resolved vacuum only MO measurement (magenta), which can be compared to DUNE stand-alone measurement (green). Given the possible uncertainties due to experiment schedules, etc, all we can say is possible $> 2030$. However, this opens for an unprecedented scenario where two as different as possible high precision MO measurements will be available to ensure the possible overall coherent of the neutrino standard phenomenology. Should discrepancies be seen, this may a smoking-gun evidence of the manifestation of new neutrino phenomenology.
  This observation implies that the JUNO based MO capability, despite its a priori humble intrinsic sensitivity, has the potential to play a critical role throughout the history of MO explorations. Indeed, the first MO fully resolved measurement is likely to depend much on the JUNO sensitivity (direct and indirectly); hence JUNO should maximise ($\Delta \chi ^2$ $\ge 9$) or maintain its yield. However, JUNO’s ultimate role aforementioned may remain relatively unaffected even by a small loss in performance, providing the overall sensitivity remains sizeable (e.g. $\Delta \chi ^2$ $\ge 7$), as illustrated in Figs. 5 and 6. This is because JUNO sensitivity could still be boosted by the LB$\nu$B experiments by their precision on $\Delta m^2_{32}$, thus sealing its legacy. There is no reason for JUNO not to perform as planned, specially given the remarkable effort for solutions and novel techniques developed, such as the dual-calorimetry, for the control and accuracy of the spectral shape^64^.
7. *LB*$\nu$*B running strategy* since both AC and DC channels drive the sensitivity of LB$\nu$B experiments, the maximal yield for a combined MO sensitivity implies a dedicated optimisation exercise, including the role of the $\delta _{\tiny \text {CP}}$ sensitivity. Indeed, as shown, the precision on $\Delta m^2_{32}$, measured via the DC channel, plays a leading role in the intrinsic MO resolution, which may even outplay the role of the AC data. So, forthcoming beam-mode running optimisation by the LB$\nu$B collaborations could, and likely should, consider the impact to MO sensitivity. In this way, if $\Delta m^2_{32}$ precision was to be optimised, this will benefit from more *neutrino mode* running, leading typically to both larger signal rate and better signal-to-background ratio. This is particularly important for T2K and HK due to their shorter baselines. For such considerations, Fig. 5 might offer some guidance.

## Conclusions

This work presents a simplified calculation tuned to the latest world neutrino data, via NuFit5.0, to study the most important minimal level inter-experiment combinations to yield the earliest possible full MO resolution (i.e. $\ge 5\sigma$). Our first finding is that the combined sensitivity of JUNO, NOvA and T2K has the potential to yield the first resolved measurement of MO with timeline between 2026-2028, tightly linked to the JUNO schedule since full data samples of both NOvA and T2K data are expected to be available from $\sim 2026$. Due to the absence of any a priori MO theory based prediction and given its intrinsic binary outcome, we noted and illustrated the benefit to articulate at least two independent and well resolved ($\ge 5\sigma$) measurements of MO. This is even more important in the light of the decisive outcome from the next generation of long baseline neutrino beams experiments. Such MO measurements could be exploited to over-constrain and test the standard oscillation model, thus opening for discovery potential, should unexpected discrepancies may manifest. However, the most profound phenomenological insight using MO phenomenology is expected to be obtained by having two different and well resolved MO measurements based on only matter effects enhanced and pure vacuum oscillations experimental methodologies. While the former is driving most of the field, the challenge was to be able to articulate the latter, so far considered as impractical. Hence, we here describe the feasible path to promote JUNO’s MO measurement to reach a robust $\ge 5\sigma$ resolution level without compromising its unique vacuum oscillation nature by exploiting the next generation long baseline neutrino beams disappearance channel’s ability to reach a precision of $\le 0.5\%$ on $\Delta m^2_{32}$.

## Supplementary Information

Supplementary Information.
