# Supplementary material for: Synergies and prospects for early resolution of the neutrino mass ordering
Source: Sci Rep. 2022 Mar 30;12:5393. doi: 10.1038/s41598-022-09111-1 (PMC8967831; doi:10.1038/s41598-022-09111-1)
Supplement: Supplementary file 1 — Supplementary Information. [file 41598_2022_9111_MOESM1_ESM.pdf]

Synergies and Prospects for Early Resolution of the Neutrino Mass Ordering: Supplementary Information

Anatael Cabrera[1,2,4], Yang Han[1,2], Michel Obolensky[1], Fabien Cavalier[2], João Coelho[2], Diana Navas-Nicolás[2], Hiroshi Nunokawa[2,8], Laurent Simard[2], Jianming Bian[3], Nitish Nayak[3], Juan Pedro Ochoa-Ricoux[3], Bedřich Roskovec[7], Pietro Chimenti[5,*], Stefano Dusini[6,*], Mathieu Bongrand[9,2], Rebin Karaparambil[9], Victor Lebrin[9], Benoit Viaud[9], Frederic Yermia[9], Lily Asquith[10], Thiago J. C. Bezerra[10], Jeff Hartnell[10], Pierre Lasorak[10], Jiajie Ling[11], Jiajun Liao[11], and Hongzhao Yu[11]

[1]APC, CNRS/IN2P3, CEA/IRFU, Observatoire de Paris, Sorbonne Paris Cité University, 75205 Paris Cedex 13, France
[2]IJCLab,, Université Paris-Saclay, CNRS/IN2P3, 91405 Orsay, France
[3]Department of Physics and Astronomy, University of California at Irvine, Irvine, California 92697, USA
[4]LNCA Underground Laboratory, CNRS/IN2P3 - CEA, Chooz, France
[5]Departamento de Física, Universidade Estadual de Londrina, 86051-990, Londrina – PR, Brazil
[6]INFN, Sezione di Padova, via Marzolo 8, I-35131 Padova, Italy
[7]Institute of Particle and Nuclear Physics, Faculty of Mathematics and Physics, Charles University, V Holešovičkách 2, 180 00 Prague 8, Czech Republic
[8]Department of Physics, Pontifícia Universidade Católica do Rio de Janeiro, Rio de Janeiro, RJ, 22451-900, Brazil
[9]SUBATECH, CNRS/IN2P3, Université de Nantes, IMT-Atlantique, 44307 Nantes, France
[10]Department of Physics and Astronomy, University of Sussex, Falmer, Brighton BN1 9QH, United Kingdom
[11]Sun Yat-sen University, NO. 135 Xingang Xi Road, Guangzhou, China, 510275
[*]Corr.Auth.: pietro.chimenti@uel.br,stefano.dusini@pd.infn.it

March 24, 2022

Introduction

This document serves as a supplementary material to the work [1]. It describes some details regarding the analyses performed in this reference.

A. Empirical Reproduction of the χ^2 Function for the LBνB-II Experiments

In this section, we shall detail how we computed the number of events for T2K and NOvA. For a constant matter density, without any approximation, appearance oscillation probability for given baseline L and neutrino energy E, can be expressed [2] as

$$\begin{aligned} P(\nu_\mu \to \nu_e) &= a_\nu + b_\nu \cos\delta_{\rm CP} + c_\nu \sin\delta_{\rm CP}, \\ P(\bar\nu_\mu \to \bar\nu_e) &= a_{\bar\nu} + b_{\bar\nu} \cos\delta_{\rm CP} + c_{\bar\nu} \sin\delta_{\rm CP}, \end{aligned} \quad (1)$$

where a_ν, b_ν, c_ν, $a_{\bar\nu}$, $b_{\bar\nu}$ and $c_{\bar\nu}$ are some factors which depend on the mixing parameters (θ_{12}, θ_{23}, θ_{13}, δm^2_{21} and Δm^2_{32}), E, L as well as the matter density. This implies that, even after taking into account the neutrino flux spectra, cross sections, energy resolution, detection efficiencies, and so on, which depend on neutrino energy, and after performing integrations over the true and reconstructed neutrino energies, the expected number of ν_e ($\bar\nu_e$) appearance events, N_{ν_e} ($N_{\bar\nu_e}$), for a given experimental exposure (running time) have also the similar $\delta_{\rm CP}$ dependence as,

$$\begin{aligned} N_{\nu_e} &= n_0 + n_c \cos\delta_{\rm CP} + n_s \sin\delta_{\rm CP}, \\ N_{\bar\nu_e} &= \bar n_0 + \bar n_c \cos\delta_{\rm CP} + \bar n_s \sin\delta_{\rm CP}, \end{aligned} \quad (2)$$

where n_0, n_c, n_s, $\bar n_0$, $\bar n_c$ and $\bar n_s$ are some constants which depend not only on mixing parameters but also on experimental setups. Assuming that background (BG) events do not depend (or depend very weakly) on $\delta_{\rm CP}$, the constant terms n_0 and $\bar n_0$ in Eq. (2) can be divided into the signal contribution and BG one as $n_0 = n_0^{\rm sig} + n_0^{\rm BG}$ and $\bar n_0 = \bar n_0^{\rm sig} + \bar n_0^{\rm BG}$, as an approximation.

In Table A1, we provide the numerical values of these coefficients which can reproduce quite well the expected number of events shown in the plane spanned by $N_{\nu_e}^{\rm obs}$ and $N_{\bar\nu_e}^{\rm obs}$, often called bi-rate plots, found in the presentations by T2K [3] and NOvA [4] at Neutrino 2020 Conference, for their corresponding accumulated data (or exposures). We show in the left panels of Figures A1 and A2, respectively, for T2K and NOvA, the bi-rate plots which were reproduced by using the values given in Table A1. Our results are in excellent agreement with the ones shown by the collaborations [3, 4].

The χ^2 function for the appearance channel (AC), for a given LBνB experiment, T2K or NOvA, which is based on the total number of events, is simply defined as follows, for each MO,

$$\chi^{2\,\mathrm{AC}}_{\mathrm{LB}\nu\mathrm{B}} \equiv \min_{s^2_{23},\,\delta_{\mathrm{CP}}} \left[\frac{(N^{\mathrm{obs}}_{\nu_e} - N^{\mathrm{theo}}_{\nu_e}(s^2_{23}, \delta_{\mathrm{CP}}))^2}{N^{\mathrm{obs}}_{\nu_e}} + \frac{(N^{\mathrm{obs}}_{\bar{\nu}_e} - N^{\mathrm{theo}}_{\bar{\nu}_e}(s^2_{23}, \delta_{\mathrm{CP}}))^2}{N^{\mathrm{obs}}_{\bar{\nu}_e}} + \chi^2_{\mathrm{pull}}(\sin^2\theta_{23}) \right], \quad (3)$$

where $N^{\mathrm{obs}}_{\nu_e}$ ($N^{\mathrm{obs}}_{\bar{\nu}_e}$) is the number of observed (or to be observed) ν_e ($\bar{\nu}_e$) events, and $N^{\mathrm{theo}}_{\nu_e}$ ($N^{\mathrm{theo}}_{\bar{\nu}_e}$) are the corresponding theoretically expected numbers (or prediction), and

$$\chi^2_{\mathrm{pull}}(\sin^2\theta_{23}) \equiv \left(\frac{\sin^2\theta^{\mathrm{true}}_{23} - \sin^2\theta_{23}}{\sigma(\sin^2\theta_{23})} \right)^2. \quad (4)$$

Note that the number of events in Eq. (3) include also background events.

	$n^{\mathrm{sig}}_0/\bar{n}^{\mathrm{sig}}_0$	$n^{\mathrm{BG}}_0/\bar{n}^{\mathrm{BG}}_0$	$n_c/\bar{n}_c$	$n_s/\bar{n}_s$
T2K ν NMO	68.6	20.2	0.2	-16.5
T2K $\bar{\nu}$ NMO	6.0	12.5	0.2	2.05
T2K ν IMO	58.1	20.2	0.7	-15.5
T2K $\bar{\nu}$ IMO	14.0	6.0	0.05	2.40
NOvAν NMO	70.0	26.8	3.2	-13.2
NOvA$\bar{\nu}$ NMO	18.7	14.0	1.3	3.7
NOvAν IMO	45.95	26.8	-3.25	-10.75
NOvA$\bar{\nu}$ IMO	26.2	14.0	-1.5	5.0

Table A1: **NOvA and T2K Oscillation Probability Empirical Parametrisation as of Neutrino 2020 Conference.** The numerical values of the factors appear in Eq. (2) are shown, which were adjusted to approximately agree with what have been presented by T2K [3] and NOvA [4]. These numbers correspond to the exposures of 2.0(1.6) $\times 10^{21}$ protons on target (POT) for ν ($\bar{\nu}$) mode of T2K and 1.4(1.3) $\times 10^{21}$ POT for ν ($\bar{\nu}$) mode of NOvA experiments. The 3 factors n^{sig}_0, n_c and n_s correspond to the case where $\sin^2\theta_{23} = 0.55$ (0.57) for T2K (NOvA) and they scale as $n^{\mathrm{sig}}_0 \propto \sin^2\theta_{23}$ and $n_c, n_s \propto \sin^2 2\theta_{23}$ as θ_{23} varies. The values of Δm^2_{32} are fixed to $\Delta m^2_{32} = 2.49(-2.46) \times 10^{-3}$ eV^2 for NMO (IMO) for T2K [3] and $\Delta m^2_{32} = 2.40(-2.44) \times 10^{-3}$ eV^2 for NMO for NOvA [4, 5].

Using the number of events given in Eq. (2) with values of coefficients given in Table A1 we performed a fit to the data recently reported by T2K at Neutrino 2020 Conference [3] just varying $\sin^2\theta_{23}$ and δ_{CP} and could reproduce rather well the $\Delta\chi^2$ presented by T2K in the same conference mentioned above, as shown in the right panel of Figure A1. We have repeated the similar exercises also for NOvA and obtained the results, shown in the right panel of Figure A2, which are reasonably in agreement with what was presented by NOvA at at Neutrino 2020 Conference [4]. In the case of NOvA the agreement is slightly worse as compared to the case of T2K. We believe that this is because, for the results shown in Figure A2, unlike the case of T2K, we did not take into account the θ_{23} constraint by NOvA (or we have set χ_{pull} in Eq. (4) equals to zero) as this information was not reported in [4].

We note that for this part of our analysis, we considered only the dependence of $\sin^2\theta_{23}$ and δ_{CP} and ignore the uncertainties of all the other mixing parameters as we are computing the number of events in an approximated way, as described above, by taking into account only the variation due to $\sin^2\theta_{23}$ and δ_{CP} with all the other parameters fixed (separately by T2K [3] and NOvA [4] collaborations) to some values which are close to the values given in Table 1 of [1].

In particular, we neglected the uncertainty of Δm^2_{32} in the LBνB AC part analysis when it is combined with JUNO plus LBνB DC part analysis to obtain our final boosted MO sensitivities. Strictly speaking, Δm^2_{32} must be varied simultaneously (in a synchronised way) in the χ^2 defined in Eq. (5) when it is combined with the χ^2 defined in Eq. (14). However, in our analysis, we simply add $\Delta\chi^2$ obtained from our simplified LBνB AC simulation which ignored Δm^2_{32} uncertainty, to the JUNO's boosted χ^2 (described in detail in the Appendix C). This can be justified by considering that a variation of Δm^2_{32} of about $\sim 1\%$ imply only a similar magnitude of variations in the appearance oscillation probabilities, which would be significantly smaller than the statistical uncertainties of LBνB-II AC mode, which are expected to reach at most the level or $\sim$5% or larger even in our future projections for T2K and NOvA.

For the MO resolution sensitivity shown in Figure 2 of [1] and used for our analysis throughout this work, we define the $\Delta\chi^2$ (labeled as $\Delta\chi^{2\,\mathrm{AC}}_{\mathrm{LB}\nu\mathrm{B}}$), as

$$\Delta\chi^{2\,\mathrm{AC}}_{\mathrm{LB}\nu\mathrm{B}}(\mathrm{MO}) \equiv \pm \min_{\sin^2\theta_{23},\,\delta_{\mathrm{CP}}} \left[\chi^{2\,\mathrm{AC}}_{\mathrm{LB}\nu\mathrm{B}}(\mathrm{IMO}) - \chi^{2\,\mathrm{AC}}_{\mathrm{LB}\nu\mathrm{B}}(\mathrm{NMO}) \right], \quad (5)$$

where +(-) sign corresponds to the case where the true MO is normal (inverted), and $\chi^{2\,\mathrm{AC}}_{\mathrm{LB}\nu\mathrm{B}}$ is computed as defined in Eq. (3) but with $N^{\mathrm{obs}}_{\nu_e(\bar{\nu}_e)}$ replaced by the theoretically expected ones for given values of assumed true values of θ_{23} and δ_{CP}. In practice, since we do not consider the effect of fluctuation for this part of our analysis, $\chi^{2\,\mathrm{AC}}_{\mathrm{LB}\nu\mathrm{B\ min}} = 0$ by construction for true MO. We

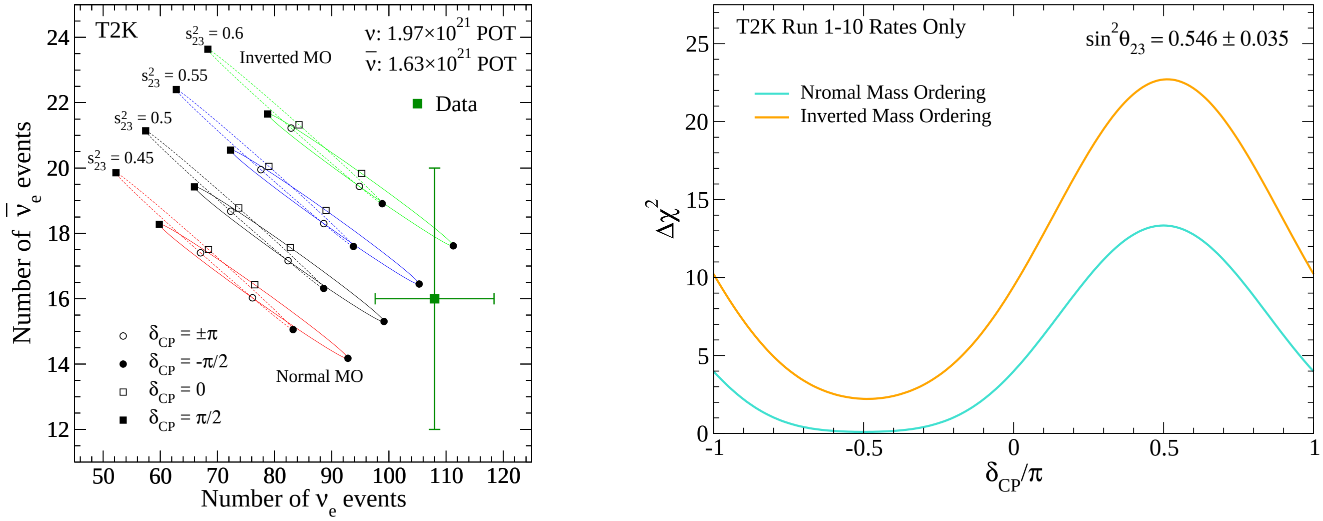

Figure A1: **Reproduction of T2K Bi-Rate and CP Sensitivity Results as of Neutrino 2020 Conference.** Left panel is the bi-rate plot which shows simultaneously the expected number of events for ν_e and $\bar{\nu}_e$ by varying continuously the values of $\delta_{\rm CP}$ from $-\pi$ to π, indicated by the solid and dashed curves (ellipses) for 4 different values of $s^2_{23} = \sin^2\theta_{23}$ indicated in the legend for the exposure of 2.0(1.6) $\times 10^{21}$ POT for neutrino (anti-neutrino) mode. The point corresponding to the latest T2K data reported at Neutrino 2020 Conference [3] is indicated by the solid dark green square with 1σ error bars. Right panel shows the $\Delta\chi^2$ obtained by fitting the data using the χ^2 function given in Eq. (3).

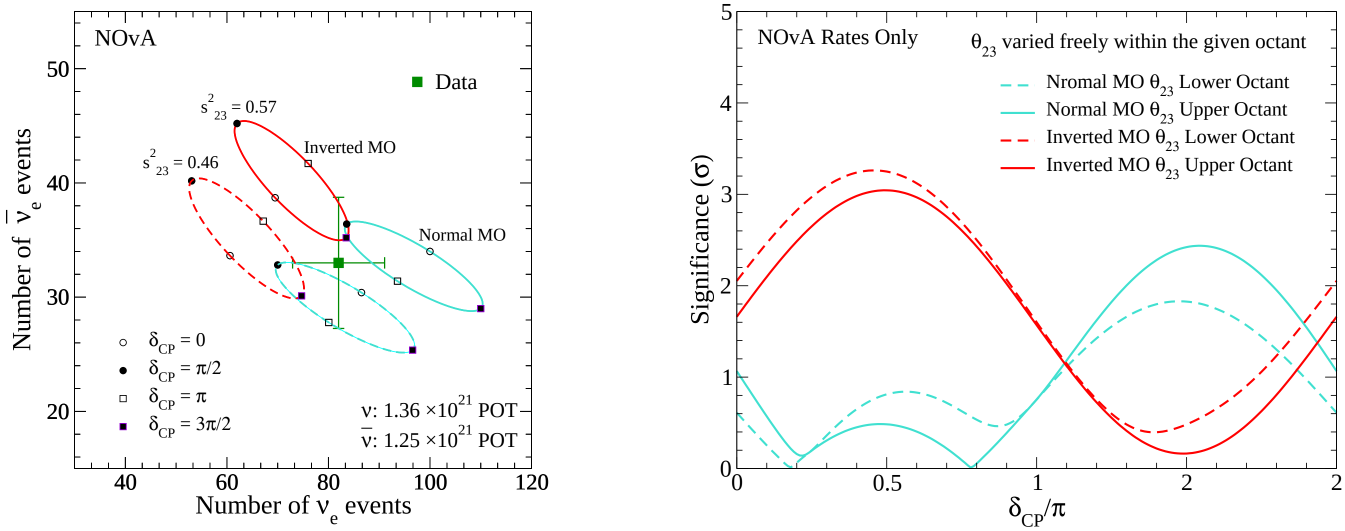

Figure A2: **Reproduction of NOvA Bi-Rate and CP Sensitivity Results as of Neutrino 2020 Conference.** Left panel is the bi-rate plot which shows simultaneously the expected number of events for ν_e and $\bar{\nu}_e$ by varying continuously the values of $\delta_{\rm CP}$ from $-\pi$ to π, indicated by the solid and dashed curves (ellipses) for 4 different values of $s^2_{23} = \sin^2\theta_{23}$ indicated in the legend for the exposure of 1.4 (1.3) $\times 10^{21}$ POT for neutrino (anti-neutrino) mode. The point corresponding to the latest NOvA data reported at Neutrino 2020 Conference [4] is indicated by the solid dark green square with 1σ error bars. Right panel shows the significance $\sqrt{\Delta\chi^2}$ obtained by fitting the data using the χ^2 function given in Eq. (3) but by setting $\chi^2_{\rm pull}(\sin^2\theta_{23})$ =0.

note that when T2K and NOvA are combined, some enhancement of sensitivities in the positive (negative) $\delta_{\rm CP}$ region for NMO (IMO) occur (see light green curves in Figure 6 of [1]. This is because that in these $\delta_{\rm CP}$ ranges, T2K and NOvA data can not be simultaneously fitted very well by using the common $\delta_{\rm CP}$ for the wrong MO, leading to an increase of $\Delta\chi^2$.

For simplicity, for our future projection, we simply increase by a factor of 3 both T2K and NOvA exposures, to the coefficients given in Table A1 for both ν and $\bar{\nu}$ channels. This corresponds approximately to 8.0 (6.4)$\times 10^{21}$ POT for T2K $\nu\,(\bar{\nu})$ mode and to 4.1 (3.8)$\times 10^{21}$ POT for NOvA $\nu\,(\bar{\nu})$ mode, to reflect roughly the currently considered final exposures for T2K [6] ($\simeq 10\times 10^{21}$ POT in total for ν and $\bar{\nu}$) and NOvA [5] ($\simeq 3.2\times 10^{21}$ POT each for ν and $\bar{\nu}$). This approach implies that our calculation does not consider future unknown optimisations on the $\nu\,(\bar{\nu})$ mode running.

B. LBνB Disappearance MO Sensitivity

In the upper panel of Figure A3, we show the 4 curves of survival oscillation probabilities, $P(\nu_\mu \to \nu_\mu)$ and $P(\bar{\nu}_\mu \to \bar{\nu}_\mu)$ for NMO and IMO, which were obtained by using the best fitted parameters in NuFit5.0 given in Table 1 of [1] for the baseline corresponds to NOvA($L=810$ km) and with the matter density of $\rho = 2.8$ g/cm^3. The NMO and IMO cases are shown, respectively, by blue and red colours whereas the cases for ν and $\bar{\nu}$ are shown, respectively, by solid thin and dashed thick curves. We observe that all of these 4 curves coincide very well with each other, so differences are very small. In the lower panel of the same Figure A3, we show the differences of these curves, between ν and $\bar{\nu}$ channels for both NMO and IMO, as well as between NMO and IMO for both ν and $\bar{\nu}$, as indicated in the legend. We observe that the differences of these oscillation probabilities are $\leq$1% for the energy range relevant for NOvA.

Two points can be highlighted. First, the fact that the differences between neutrino and anti-neutrino are quite small implies that the matter effects are very small in these channels, hence determining MO by using matter effects based only on LBνB DC would be almost impossible. And second, the fact that the curves for NMO and IMO agree very well implies that the absolute values of the effective mass squared differences, called $\Delta m^2_{\mu\mu}$, defined in Eq. (11) in Appendix D, which correspond to NMO and IMO cases, should be similar. Indeed, by using the values given in Table 1 of [1], we obtain $\Delta m^2_{\mu\mu} = 2.422(-2.431)\times 10^{-3}{\rm eV}^2$ for NMO (IMO) exhibiting a small $\sim 0.4\%$ difference. In other words, for each channel, ν and $\bar{\nu}$, there are two *degenerate* solutions, one corresponds to NMO and the other, to IMO, which give in practice the same survival probabilities. We stress that this degeneracy can not be resolved by considering LBνB experiment with DC alone.

C. Analytic Understanding of Synergy between JUNO and LBνB based experiments

In this section, we shall detail the relation between true and false Δm^2_{32} solutions in the case of JUNO and LBνB, as they are different. This difference is indeed exploited as the main numerical quantification behind the $\Delta\chi^2_{\rm BOOST}$ term which was schematically illustrated in Figure 3 of [1] and will be further quantified in Figure A4 to be shown in this appendix.

C.1 JUNO Relation between True-False Δm^2_{32}

The $\bar{\nu}_e \to \bar{\nu}_e$ survival probability in vacuum can be expressed as [7]

$$P_{\bar{\nu}_e\to\bar{\nu}_e} = 1 - c_{13}^4 \sin^2 2\theta_{12} \sin^2\Delta_{21} - \frac{1}{2}\sin^2 2\theta_{13} \times \left[1 - \sqrt{1-\sin^2 2\theta_{12}\sin^2\Delta_{21}}\cos(2|\Delta_{ee}| \pm \phi)\right], \quad (6)$$

where the notation $c_{ij} \equiv \cos\theta_{ij}$ and $s_{ij} \equiv \sin\theta_{ij}$ is used, and $\Delta_{ij} \equiv \Delta m^2_{ij} L/4E$, L and E are, respectively, the baseline and the neutrino energy, and the effective mass squared difference Δm^2_{ee} is given by [8]

$$\Delta m^2_{ee} \equiv c_{12}^2 \Delta m^2_{31} + s_{12}^2 \Delta m^2_{32} = \Delta m^2_{32} + c_{12}^2 \Delta m^2_{21}, \quad (7)$$

and ϕ is given by

$$\tan\phi = \frac{c_{12}^2 \sin(2 s_{12}^2 \Delta_{21}) - s_{12}^2 \sin(2 c_{12}^2 \Delta_{21})}{c_{12}^2 \sin(2 s_{12}^2 \Delta_{21}) + s_{12}^2 \sin(2 c_{12}^2 \Delta_{21})}, \quad (8)$$

where $\phi \simeq 0.36$ radian $\simeq 0.11\pi$ for $s_{12}^2 = 0.304$ and $\delta m^2_{21} = 7.42\times 10^{-5}$ eV^2. The +(-) sign in front of ϕ in Eq. (6) corresponds to the normal (inverted) mass ordering.

Upon data analysis, JUNO will obtain two somewhat different values of Δm^2_{32} corresponding to NMO and IMO, which we call $\Delta m^{2\ \rm NMO}_{32\ \rm JUNO}$ and $\Delta m^{2\ \rm IMO}_{32\ \rm JUNO}$ where one of them should correspond (or closer) to the true solution. It is expected that by considering $\Delta^{\rm NMO}_{ee} + \phi = \Delta^{\rm IMO}_{ee} - \phi$, they are approximately related by

$$\Delta m^{2\ \rm IMO}_{32\ \rm JUNO} \simeq -\Delta m^{2\ \rm NMO}_{32\ \rm JUNO} - 2c_{12}^2 \delta m^2_{21} - \delta m^2_\phi, \quad (9)$$

where the approximated value of δm^2_ϕ can be estimated by choosing the average representative energy of reactor neutrinos ($\sim$4 MeV) as

$$\delta m^2_\phi \equiv \frac{4E}{L}\phi \simeq 2.1\times 10^{-5}\left(\frac{E}{4\ \rm MeV}\right){\rm eV}^2. \quad (10)$$

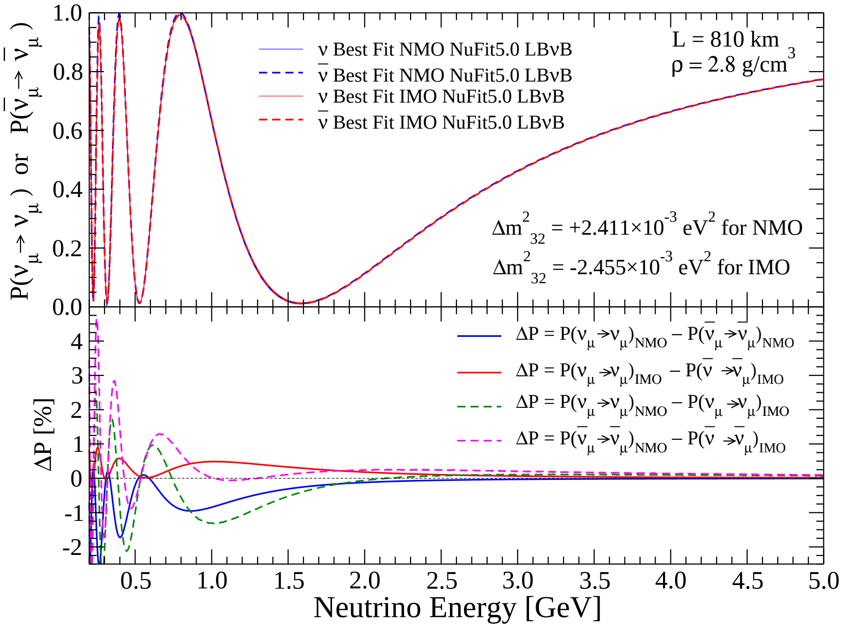

Figure A3: **LBνB Survival Probability Mass Ordering Dependence.** In the upper panel, the $\nu_\mu \to \nu_\mu$ and $\bar{\nu}_\mu \to \bar{\nu}_\mu$ survival probabilities computed by using the mixing parameters found in Table 1 of [1] are shown for NMO (solid and dashed red curves) and IMO (solid and dashed blue curves) as a function of neutrino energy, as indicated in the legend. In the lower panel, the differences of these probabilities are shown in percent.

We found that for a given assumed true value of $\Delta m^2_{32} = 2.411 \times 10^{-3}$ eV^2 (corresponding to NMO), we can reproduce very well the false value of $\Delta m^2_{32} = -2.53 \times 10^{-3}$ eV^2 (corresponding to IMO) obtained by a χ^2 fit if we use $E = 4.4$ MeV in Eqs. (9) and (10). The relation between true and false Δm^2_{32} for JUNO is illustrated by the vertical black dashed and black solid lines in Figure A4 (b) and (d).

C.2 LBνB Relation between True-False Δm^2_{32}

For LBνB experiments like T2K and NOvA the L/E are such that $|\Delta_{31}| \sim |\Delta_{32}| \sim \pi/2$. From the disappearance channels $\nu_\mu \to \nu_\mu$ and $\bar{\nu}_\mu \to \bar{\nu}_\mu$, it is possible to measure precisely the effective mass squared difference $\Delta m^2_{\mu\mu}$ whose value is independent of the MO. In terms of fundamental mixing and oscillation parameters, $\Delta m^2_{\mu\mu}$ can be expressed, with very good approximation, as [8],

$$\Delta m^2_{\mu\mu} \equiv \Delta m^2_{32} + (s^2_{12} + \cos\delta_{\rm CP} s_{13} \sin 2\theta_{12} \tan\theta_{23}) \delta m^2_{21}. \quad (11)$$

From this relation, one can extract two possible values of Δm^2_{32} corresponding to two different MO as

$$\Delta m^{2\;\rm MO}_{32\;\rm LB\nu B} = +(-)|\Delta m^2_{\mu\mu}| - (s^2_{12} + \cos\delta^{\rm MO}_{\rm CP} s^{\rm MO}_{13} \sin 2\theta_{12} \tan\theta^{\rm MO}_{23}) \delta m^2_{21}, \quad (12)$$

where superscript MO implies either NMO or IMO, and + and - sign correspond, respectively, to NMO and IMO. Note that the best fitted values for the mixing and oscillation parameters, with the exception of solar parameters θ_{12} and δm^2_{21}, can be different in the NMO and IMO scenarios. Eq. (12) can be rewritten as

$$\begin{aligned} \Delta m^{2\;\rm IMO}_{32_{\rm LB\nu B}} &= -\Delta m^{2\;\rm NMO}_{32_{\rm LB\nu B}} - \delta m^2_{21} \left\{ 2s^2_{12} + \sin 2\theta_{12} \right. \\ &\left. (\cos\delta^{\rm NMO}_{\rm CP} s^{\rm NMO}_{13} \tan\theta^{\rm NMO}_{23} + \cos\delta^{\rm IMO}_{\rm CP} s^{\rm IMO}_{13} \tan\theta^{\rm IMO}_{23}) \right\} \\ &\simeq -\Delta m^{2\;\rm NMO}_{32_{\rm LB\nu B}} - \delta m^2_{21} \left\{ 2s^2_{12} + \sin 2\theta_{12} \right. \\ &\left. \times s_{13} \tan\theta_{23} (\cos\delta^{\rm NMO}_{\rm CP} + \cos\delta^{\rm IMO}_{\rm CP}) \right\}, \quad (13) \end{aligned}$$

where in the last line of the above equation, some simplifications were considered based on the fact that best fitted values of $\sin^2\theta_{13}$ and $\sin^2\theta_{23}$ in recent global analysis [9] are similar for both MO solutions. By using the relation given in Eq. (13), for a given assumed true value of Δm^2_{32} (common for all experiments) we obtain the yellow colour bands shown in Figure A4 (b) and (d).

C.3 Boosting Synergy Estimation

The extra synergy for MO determination sensitivity by combining JUNO and LBνB DC can be achieved thanks to the *mismatch* (or disagreement) of the fitted Δm^2_{32} values for the wrong MO solutions between these two

types of experiments. For the correct MO, Δm^2_{32} values measured by different experiments should agree with each other within the experimental uncertainties. But for those values which correspond to the wrong MO do not agree. The difference can be quantified and used to enhance the sensitivity.

Following the procedure described in [10, 11], we include to the JUNO analysis the external information on the external information on Δm^2_{32} from LBνB with an additional pull term as

$$\chi^2 = {\chi^2}_{\text{JUNO}} + \left(\frac{\Delta m^2_{32} - \Delta m^{2\ \text{NMO or IMO}}_{32\,\text{LB}\nu\text{B}}}{\sigma(\Delta m^2_{32})_{\text{LB}\nu\text{B}}} \right)^2 , \quad (14)$$

where χ^2_{JUNO} implies the χ^2 function for JUNO alone computed in a similar fashion as in [11], $\sigma(\Delta m^2_{32})_{\text{LB}\nu\text{B}}$ is the experimental uncertainty on Δm^2_{32} achieved by LBνB based experiments. As typical values in this paper, we consider 3 cases $\sigma(\Delta m^2_{32})_{\text{LB}\nu\text{B}} = 1$, 0.75 and 0.5%.

In order to take into account the possible fluctuation of the central values of the measured $\Delta m^2_{32\,\text{LB}\nu\text{B}}$ we define the extra boosting $\Delta\chi^2$ due to the synergy of JUNO and LBνB based experiments as the difference of χ^2 defined in Eq. (14) for normal and inverted MO as,

$$\Delta\chi^2_{\text{boost}} \equiv \pm \left(\chi^2_{\text{IMO}} - \chi^2_{\text{NMO}} \right) , \quad (15)$$

where +(-) sign corresponds to the case where the true MO is normal (inverted). Note that in our simplified phenomenological approach (based on the future simulated JUNO data), for the case with no fluctuation, by construction, $\chi^2_{\text{NMO (IMO)}} = 0$ for NMO (IMO).

Let us try to see how the boosting will be realized by applying our discussion to JUNO and T2K for illustration. In Figure A4 for the cases where the true MO is normal in the panel (a) and inverted in the panel (c) we show by the solid (dashed) black curve $\Delta\chi^2$ for JUNO alone case for true (false) MO. The difference of $\Delta\chi^2$ between true and false MO is 9 if only JUNO is considered implying that the false MO (indicated by the dashed curves) can be rejected at 3σ. On the other hand, let us assume the case where T2K can determine $|\Delta m^2_{32}|$ with 1% uncertainty, and the corresponding $\Delta\chi^2$ curves are given by the solid (dashed) blue curves for true (false) MO in the same plots, rejecting the wrong MO only at 2σ by T2K alone. If we combine JUNO and T2K following the procedure described in this section, the resulting $\Delta\chi^2$ are given by the solid (dashed) red curves for true (false) MO, rejecting the wrong MO with more than 4σ for both NMO and IMO.

The large (~ 10) increase of the combined $\Delta\chi^2$ for the wrong MO fit comes from the mismatch of the false $|\Delta m^2_{32}|$ values between JUNO (black dashed line) and T2K (yellow colour bands) shown in the panels (b) and (d) of Figure A4. This is nothing the boosting effect, which can be analytically understood and quantified as follows.

Suppose that we try to perform a χ^2 fit assuming the wrong MO. Let us first assume that $\sigma(\Delta m^2_{32})_{\text{JUNO}} \ll \sigma(\Delta m^2_{32})_{\text{LB}\nu\text{B}}$ and no fluctuation for simplicity (i.e. ${\chi^2}_{\text{true MO}} = 0$). The first term in Eq. (14), ${\chi^2}_{\text{JUNO}}$, forces to drive the fitted value of Δm^2_{32} very close to the *false* one favoured by JUNO or $\Delta m^{2\ \text{false}}_{32\,\text{JUNO}}$ (otherwise, ${\chi^2}_{\text{JUNO}}$ value increases significantly). Then the extra increase of χ^2 is approximately given by the second term in Eq. (14) with Δm^2_{32} replaced by $\Delta m^{2\ \text{false}}_{32\,\text{JUNO}}$,

$$\begin{aligned} \Delta\chi^2_{\text{boost}} &\sim \left[\frac{\Delta m^{2\ \text{false}}_{32\,\text{JUNO}} - \Delta m^{2\ \text{false}}_{32\,\text{LB}\nu\text{B}}}{\sigma(\Delta m^2_{32})_{\text{LB}\nu\text{B}}} \right]^2 , \\ &\sim \left[\frac{\delta m^2_\phi + 2\delta m^2_{21}(\cos 2\theta_{12} - \sin 2\theta_{12} s_{13} \tan\theta_{23} \cos\delta_{\text{CP}})}{\sigma(\Delta m^2_{32})_{\text{LB}\nu\text{B}}} \right]^2 \\ &\sim 4, 9, 16 \text{ , respectively, for } \delta_{\text{CP}} = 0, \pm\pi/2, \pm\pi, \end{aligned} \quad (16)$$

where the numbers in the last line were estimated for $\sigma(\Delta m^2_{32})_{\text{LB}\nu\text{B}} = 1\%$. The case where $\delta_{\text{CP}} = \pm\pi/2$ and $\Delta\chi^2_{\text{boost}} \sim 9$ can be directly compared with more precise results shown in Figure 4(a) of [1], see the blue solid curve at $\delta^{\text{true}}_{\text{CP}} = \pm\pi/2$ which gives $\Delta\chi^2_{\text{boost}} \sim 8$ which is in rough agreement. The expression in Eq. (16) is in agreement with the one given in Eq. (18) of [10] apart from the term δm^2_ϕ which is not so large.

D. Full 3 ν versus effective 2ν formulation

In the previous discussions found in [10, 11], in order to demonstrate the boosting synergy effect between JUNO and LBνB experiments, the effective mass squared differences Δm^2_{ee} and $\Delta m^2_{\mu\mu}$, defined respectively, in Eqs. (7) and (11) originally found in [8] were used. While we used these parameters in some intermediate steps of our computations, as described in Appendix C, we did not use these parameters explicitly in our combined χ^2 describing the extra synergy between JUNO and LBνB (DC) based experiments defined in Eq. (14), as well as in the final sensitivity plots presented in this paper. The main advantage of using these effective mass squared differences is that no a priori assumptions have to be made about any other parameters not accessible by JUNO, in particular, CP phase (δ_{CP}), whereas by using them, one must specify explicitly the δ_{CP} value as done in Ref. [10].

In order to check the consistency between our work and previous studies, we have explicitly verified that the results do not depend on the parameters used in

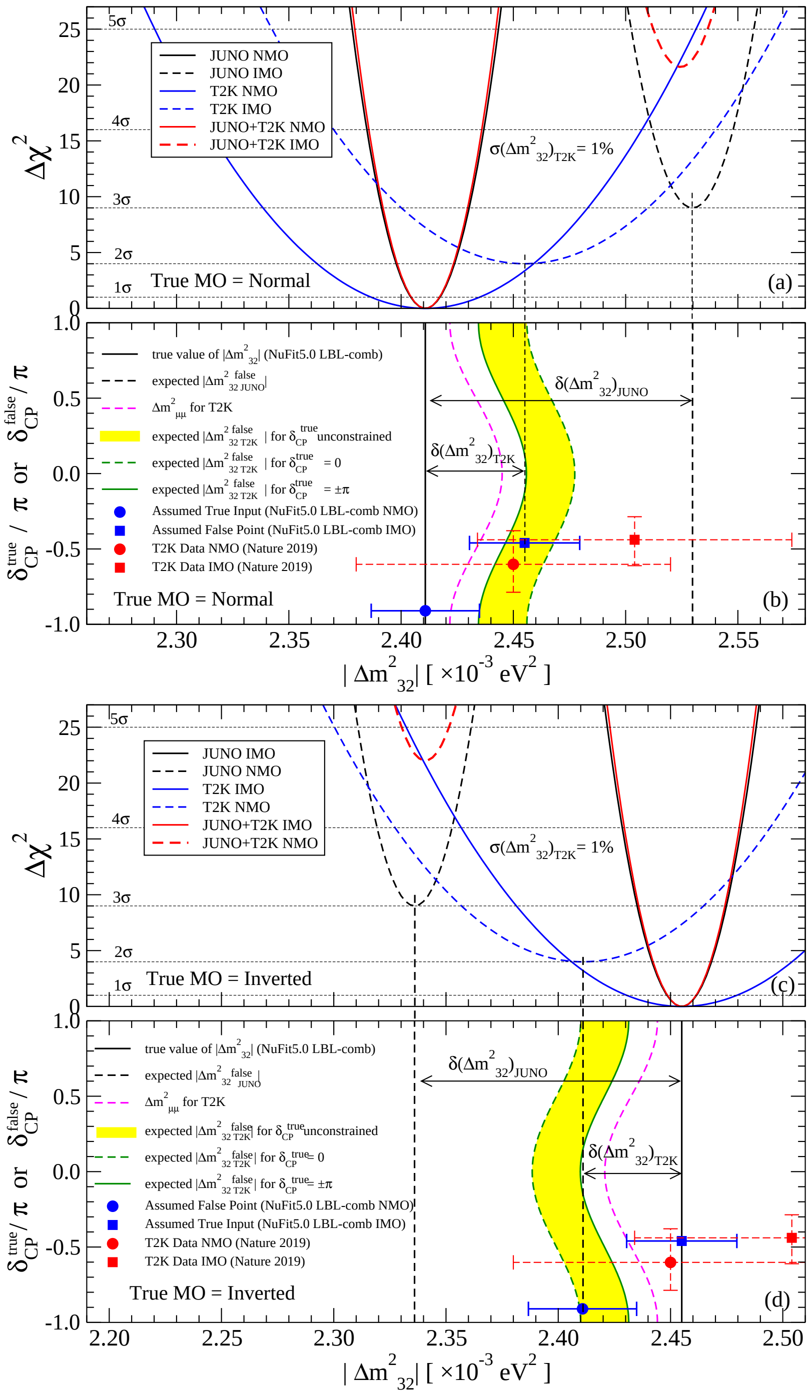

Figure A4: **JUNO & LBνB Mass Ordering Synergy.** The behaviours of $\Delta\chi^2$ terms (parabolas) are shown as a function of $|\Delta m^2_{32}|$ for JUNO (black), T2K (or NOvA), and their enhanced combination (red). The $\Delta m^{2\;\text{true}}_{32}$ is fixed to the NuFit5.0 best value shown, respectively, in panels (a) and (c) for NMO and IMO. The extra gain in $\Delta\chi^2_{\text{BOOST}}$ discrimination numerically originates from the fact that the true $|\Delta m^2_{32}|$ solutions should match between JUNO (solid black vertical line) and LBνB (solid blue vertical circle); hence the false solutions (dashed vertical lines) must differ. Panels (b) and (d) illustrate this origin. The relation between true-false Δm^2_{32} solutions is different and complementarity for JUNO and LBνB experiments. The difference is large ($\approx 1.5{\times}\delta m^2_{21}$) for JUNO. Instead, LB$\nu$B exhibits a smaller difference that modulates with δ_{CP}. So, the relative difference between $\Delta m^{2\;\text{false}}_{32\;\text{JUNO}}$ and $\Delta m^{2\;\text{false}}_{32\;\text{LB}\nu\text{B}}$ is maximal (minimal) for the δ_{CP}-conserving $\pm\pi$ (0) value. Hence, $\Delta\chi^2_{\text{BOOST}}$ depends on δ_{CP} , and ambiguity arises (yellow band) from the a priori different values of δ_{CP} for the true or false solutions. The T2K data (red points) contrasts the precision on $|\Delta m^2_{32}|$ now [12] compared to needed scenarios $\leq$1.0% scenario (blue points and parabolas). The precision of each contribution is indicated by the parabolas' width, where JUNO is fixed to the nominal value [11].

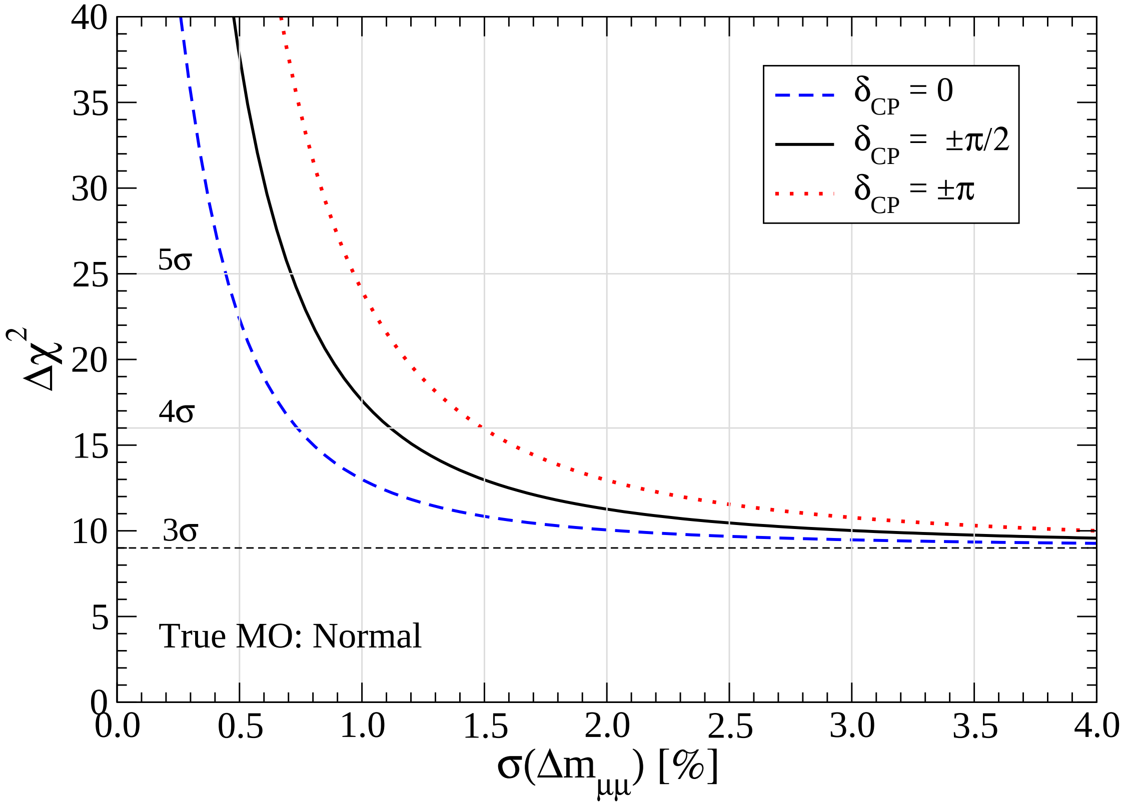

Figure A5: **$\Delta\chi^2$(JUNO⊕LBνB-DC) as a Function of the Precision of $\Delta m^2_{\mu\mu}$.** Expected MO sensitivity to be obtained by JUNO with external information of $\Delta m^2_{\mu\mu}$ coming from LBνB experiments following the procedure described in [10, 11], are shown as a function of the precision of $\Delta m^2_{\mu\mu}$ for $\cos\delta_{\rm CP} = \pm 1$ and 0. This plot is similar to Figure 7 in [10], once upgraded to the latest global data inputs [9]. We observe that they are consistent with each other, if the curves for the $\delta_{\rm CP}$ values of 0° (blue) and 180° (red) were interchanged, as a result of a typo in the legend of [10].

the analysis and in the presentation of the final results, provided that that comparisons are done properly. In Figure A5, we show $\Delta\chi^2$(JUNO⊕LBνB-DC) computed by using explicitly $\Delta m_{\mu\mu}$ (instead of using Δm^2_{32}) in our χ^2 analysis as done in [10, 11], as a function of the precision of $\Delta m^2_{\mu\mu}$. There is general good agreement with the result shown in Figure 7 of [10], if $\delta_{\rm CP}$ curves for 0° and 180° were interchanged, as described in Figure A5.

References

[1] Anatael Cabrera et al. Synergies and Prospects for Early Resolution of the Neutrino Mass Ordering. https://doi.org/10.1038/s41598-022-09111-1, 2022.

[2] Keiichi Kimura, Akira Takamura, and Hidekazu Yokomakura. Exact formulas and simple CP dependence of neutrino oscillation probabilities in matter with constant density. *Phys. Rev. D*, 66:073005, 2002.

[3] Talk presented by Patrick Dunne at The XXIX International Conference on Neutrino Physics and Astrophysics, Neutrino 2020, June 22 - July 2, 2020. `https://conferences.fnal.gov/nu2020/`, 2020.

[4] Talk presented by Alex Himmel at The XXIX International Conference on Neutrino Physics and Astrophysics, Neutrino 2020, June 22 - July 2, 2020. `https://conferences.fnal.gov/nu2020/`, 2020.

[5] Talk presented by Michael Baird at 40th International Conference on High Energy Physics (ICHEP2020), 28 July 2020 - 6 August 2020, Prague, 2020. `https://indico.cern.ch/event/868940/contributions/3817028/`, 2020.

[6] Atsuko Ichikawa, private communication.

[7] Hisakazu Minakata, Hiroshi Nunokawa, Stephen J. Parke, and Renata Zukanovich Funchal. Determination of the Neutrino Mass Hierarchy via the Phase of the Disappearance Oscillation Probability with a Monochromatic $\bar{\nu}_e$ Source. *Phys. Rev. D*, 76:053004, 2007. [Erratum: Phys.Rev.D 76, 079901 (2007)].

[8] Hiroshi Nunokawa, Stephen J. Parke, and Renata Zukanovich Funchal. Another possible way to determine the neutrino mass hierarchy. *Phys. Rev. D*, 72:013009, 2005.

[9] Ivan Esteban, M. C. Gonzalez-Garcia, Michele Maltoni, Thomas Schwetz, and Albert Zhou. The fate of hints: updated global analysis of three-flavor neutrino oscillations. *JHEP*, 09:178, 2020.

[10] Yu-Feng Li, Jun Cao, Yifang Wang, and Liang Zhan. Unambiguous Determination of the Neutrino Mass Hierarchy Using Reactor Neutrinos. *Phys. Rev. D*, 88:013008, 2013.

[11] Fengpeng An et al. Neutrino Physics with JUNO. *J. Phys. G*, 43(3):030401, 2016.

[12] K. Abe et al. Constraint on the matter–antimatter symmetry-violating phase in neutrino oscillations. *Nature*, 580(7803):339–344, 2020. [Erratum: Nature 583, E16 (2020)].
